# Integrating Molecular Docking and Electrophysiology Reveals Sesquiterpenes as Candidate Attractants for *Ceratitis capitata* Wiedemann (Diptera: Tephritidae)

**DOI:** 10.3390/insects17030251

**Published:** 2026-02-27

**Authors:** Daniela Ordaz-Pérez, Julio C. Rojas, David Alavez-Rosas

**Affiliations:** 1 Laboratorio de Taxonomía, Programa Operativo de Moscas, Planta MOSCAFRUT, Metapa de Domínguez 30826, Chiapas, Mexico; daniela.ordaz.i@senasica.gob.mx; 2 Grupo de Manejo de Plagas y Vectores de Enfermedades, Departamento de Ecología de Artrópodos y Manejo de Plagas, El Colegio de la Frontera Sur (ECOSUR), Carretera Antiguo Aeropuerto Km. 2.5, Tapachula 30700, Chiapas, Mexico; 3 Grupo de Ecología Química, Departamento de Ecología de Artrópodos y Manejo de Plagas, El Colegio de la Frontera Sur (ECOSUR), Carretera Antiguo Aeropuerto Km. 2.5, Tapachula 30700, Chiapas, Mexico; jrojas@ecosur.mx; 4 Grupo Colegiado de Investigación en Educación en Ciencias Químico Fármaco Biológicas y Salud Ambiental, Facultad de Ciencias Químicas, Benemérita Universidad Autónoma de Chiapas, Carretera a Puerto Madero Km. 1.5, Tapachula 30792, Chiapas, Mexico

**Keywords:** lures, medfly, integrated pest management, reverse chemical ecology

## Abstract

The Mediterranean fruit fly (*Ceratitis capitata*) is a globally invasive pest that affects a wide range of fruit and vegetable crops. Identifying cost-effective attractants is essential for sustainable integrated pest management (IPM). This study explored whether molecular docking, combined with electrophysiological recordings, can help prioritize structurally diverse compounds with potential relevance to medfly olfaction. We assessed the predicted interactions of more than 100 attractant-related and semiochemical compounds, including multiple stereoisomers, with 14 odorant-binding proteins (OBPs) and four odorant receptors (ORs). Trimedlure served as a benchmark ligand. Docking suggested that several sesquiterpenes may interact favorably with subsets of OBPs and ORs, although these predictions require biochemical validation. A small set of compounds with high predicted affinity, readily available in the laboratory, was further examined using electroantennography (EAG), which confirmed that selected sesquiterpenes elicited peripheral antennal activation in irradiated males. Overall, our results demonstrate the utility of computational screening as an exploratory tool for prioritizing candidate ligands and generating hypotheses about chemosensory processing in *C. capitata*. Integrating molecular modeling with biochemical and behavioral validation is a promising approach to developing next-generation IPM attractants.

## Simple Summary

The Mediterranean fruit fly is one of the most harmful pests of fruit crops worldwide, and its management relies heavily on effective attractants. In this study, we combined computer simulations with antennal response assays to identify natural scent compounds that elicit strong responses in flies. We evaluated more than 100 molecules and found that several plant-derived sesquiterpenes activated the flies’ antennae at levels comparable to those of the commercial lure trimedlure. These results suggest that sesquiterpenes could serve as affordable, environmentally friendly alternatives for developing new attractants to improve the monitoring and control of this major agricultural pest.

## 1. Introduction

The Mediterranean fruit fly (medfly), *Ceratitis capitata*, has a significant global economic impact, as its larvae infest more than 300 fruit and vegetable species [1]. Mass trapping has proven effective in Integrated Pest Management (IPM) programs for the medfly [2]. Commercial attractants have successfully developed as lures for medfly captures. The most used are: (1) the three-component biolure, a female-targeted lure for the medfly composed of ammonium acetate, putrescine, and trimethylamine; (2) Ceratrap, an enzymatically hydrolyzed animal protein, mainly comprising acetic acid and nitrogenated compounds [3]; and (3) trimedlure, the standard male-targeted medfly attractant, which is composed of a synthetic mixture of stereoisomers of tert-butyl-(4)-chloro-2-methylcyclohexane-1-carboxylate and tert-butyl-(5)-chloro-2-methylcyclohexane-1-carboxylate [3]. Additionally, naturally occurring plant-based compounds and essential oils have been extensively investigated for their ability to attract the medfly [4,5,6,7]. For instance, α-copaene is 2–5 times as attractive as trimedlure to the medfly [1,8]. Moreover, linalool, α-pinene, caryophyllene, and other sesquiterpenes have been reported as promising attractants for the medfly [6,7]. Conversely, male-produced pheromone components have been evaluated and validated through electrophysiological and behavioral studies as promising attractants [9,10]. Among these, (*E*,*E*)-α-farnesene and α-copaene are particularly important, as they form part of the pheromone of wild males [11,12]. However, there is ongoing debate about the potential adverse effects of attractants on non-target organisms, including beneficial ones [13].

Insects, including the medfly, use chemical signals and cues to communicate during all interactions with the environment; they primarily communicate chemically through olfaction. This process involves recognizing and discriminating olfactory ligands via proteins located mainly in the antennae [14,15]. Insect antennae bear sensory structures known as *sensilla*, which vary in morphology, including hair-like forms. These *sensilla* (singular: *sensillum*) are essential for environmental sensing by detecting odors [16]. The surface of each sensillum is covered with tiny pores through which odorants pass and dissolve in a fluid called sensillum lymph, which bathes the olfactory receptor neurons (ORNs) housed in a given sensillum. The odorant receptor (OR) proteins expressed by the ORNs function as odor-gated ion channels [16,17]. To transport odorant molecules to their ORs in the lymph, insects use soluble receptors such as odorant-binding proteins (OBPs). These small, water-soluble proteins bind odorant molecules and deliver them to their corresponding ORs [15,16]. The interaction of odorant molecules with ORs either increases or decreases the basal firing rate of the ORNs. This neuronal activity, in the form of action potentials, eventually conveys, after processing in the brain, the insect’s behavioral response [15,16,18].

Although insects have other sensory systems, olfaction remains critical; regarding the medfly, some OBPs have been elucidated for both sexes and distributed in all body parts; among these, the OBPs distributed predominantly in the main olfactory organs, i.e., antennae and maxillary palps are Ccapobp19a, Ccapobp19b, Ccapobp19d-1, Ccapobp28a, Ccapobp44a, Ccapobp49a, Ccapobp56d, Ccapobp56h, Ccapobp69a, Ccapobp83b, Ccapobp84a-2, Ccapobp99a, Ccapobp99c, Ccapobp99d [19,20,21]. Additionally, four putative ORs (CcOr7a, CcOr59b, CcOr83b, and CcOr85b) have been linked to trichoid sensilla and function as general odorant receptors that detect plant volatiles and kairomones in Tephritidae and related Diptera. However, their specific role in pheromone detection has not been experimentally confirmed [10,22]. Studies on the affinity of compounds for OBPs and ORs have emerged as promising tools for identifying relevant candidates to test as potential attractants that are effective, economically viable, and sustainable [9,17,23]. These studies have indicated the potential of certain compounds [9,10].

This study investigates whether computational screening can identify structurally diverse attractant-related and semiochemical compounds with potential behavioral relevance in *C. capitata*. Predicted interactions between over 100 compounds, including multiple stereoisomers, and 14 OBPs and 4 ORs are assessed, and the antennal detection of selected candidates is evaluated using electroantennography (EAG). Because *C. capitata* is a quarantined pest eradicated from Mexico, experimental work is restricted to irradiated sterile males. Accordingly, all electrophysiological assays were conducted on males. This design allowed us to evaluate whether computational screening can prioritize structurally diverse ligands with potential behavioral relevance, providing a basis for subsequent biochemical validation and behavioral testing.

## 2. Materials and Methods

### 2.1. Protein Molecular Models

Medfly OBPs and ORs molecular models were obtained from the UniProt database, and the gene or amino acid sequence was obtained from the GenBank database. The molecular model was constructed through sequence homology modeling with the SWISS-Prot server [24]. OBP and OR structures were obtained either directly from available predicted 3D models (UniProt and AlphaFold) or by homology modeling when no structural model was available. All models were subsequently energy-minimized and validated before docking. The molecular models were obtained in PDB files and subjected to energy minimization with the YASARA server [25].

Molecular models were retrieved directly from UniProt: CcOr7a (W8C7P2), CcOr59b (Q5D7J4), CcOr83b (W8BXK2), and CcOr85b (W8AKN0). Similarly, several OBPs: Ccapobp56d (W8CBW7), Ccapobp44a (A0A811VG75), Ccapobp19d-1 (W8AVF6), Ccapobp56h (A0A811VEP5), Ccapobp99c (A0A811U5U8), Ccapobp84a-2 (W8B841), Ccapobp99d (W8C709), and Ccapobp69a (W8W3V2). For OBPs lacking molecular models in UniProt, homology-based modeling was performed using the SWISS-MODEL server. Specifically, Ccapobp99a (XP_004521185.1) and Ccapobp19b (XP_004525027.1) were modeled using *Anastrepha obliqua* Macquart OBP8 (78.95% similarity) and OBP19b (71.97% similarity) as templates, respectively. Ccapobp19a (XP_004525026.1) was modeled using *Drosophila melanogaster* Meigen OBP19a (61.98% similarity). Ccapobp28a (XP_004525016), Ccapobp83a (NM_001308404.1), and Ccapobp49a were modeled using three *Bactrocera dorsalis* Hendel OBPs (A0A0G3Z688, A0A034WTQ8, and A0A6I9UX31) with 80.47%, 71.97%, and 75.5% sequence similarity, respectively. The quality of all structural models was validated prior to docking: homology models were evaluated using GMQE and QMEAN Z-scores from SWISS-MODEL, AlphaFold-predicted models were assessed using per-residue pLDDT confidence scores, and all models were further validated with MolProbity (Ramachandran statistics, MolProbity scores, and clash scores).

### 2.2. Semiochemicals and Attractants

A total of 112 compounds were selected for evaluation (Supplementary Table S1), including reported medfly attractants, volatiles from fermented fruits, semiochemicals from other fruit fly species, and their corresponding stereoisomers. The semiochemical molecular models were downloaded from the PubChem database [26], and stereoisomers were generated using ChemDraw 19.0. All molecular models were energy-minimized before generating the mol2 files using Avogadro 1.2.0 [27].

### 2.3. Docking Studies

Molecular docking analyses were conducted using AutoDock Tools 1.5.6 software [28]. The OBPs and ORs were prepared by adding polar hydrogen atoms and assigning Kollman charges. The semiochemicals were prepared by removing the non-polar hydrogen atoms and adding Gasteiger charges. Blind docking was performed using a large grid box (80 × 80 × 80) covering most of the protein surface. We measured the free binding energy of each compound (in Kcal/mol) using all the OBPs and ORs. Docking energies across all 14 OBPs and 4 ORs were averaged to create a reliable ranking metric focused on prioritizing broad-spectrum candidate ligands. The goal of this method was not to determine protein-specific binding mechanisms but to minimize the impact of receptor structural differences and to identify compounds consistently predicted to bind multiple targets. Individual docking values for each OBP/OR are included in the Supplementary Material (Supplementary Table S2). Supplementary Figures S2 and S3 provide representative molecular docking poses for trimedlure C and α-copaene, illustrating their predicted interactions with OBPs and ORs.

### 2.4. Electroantennogram

Among the docked compounds, only those with binding scores lower than that of trimedlure and available for electrophysiological assays were selected for EAG validation. Irradiated, sterile 5-day-old *C. capitata* males of the Vienna 8 genetic sexing strain were obtained weekly from the Moscamed mass-rearing facility at Metapa, Chiapas, Mexico. Irradiation was performed at the pupal stage using gamma radiation, following the standard protocol for sterile male production. At 5 days of age, males are sexually mature and physiologically active, making them suitable for electrophysiological assays. Females were not included because the Vienna 8 strain is maintained as a male-only line at the facility. Approximately 100 pupae were placed in 30 × 30 × 40 cm Plexiglas cages with water and food (1:3 hydrolyzed yeast and sugar mix) and maintained at 25 ± 1 °C until adult emergence. Twenty irradiated medfly males were sampled, and their antennae were carefully removed. The base of the antenna was inserted into a reference glass capillary electrode previously filled with Ringer’s solution. Meanwhile, the distal end was inserted into the tip of the glass recording capillary electrode. The signals generated by the antenna were passed through a high-impedance amplifier (NL 1200; Syntech GmbH, Buchenbach, Germany) and displayed using Syntech EAG software V2.1 (Ockenfels Syntech GmbH, Buchenbach, Germany) for processing EAG signals. A stimulus flow controller (CS-05; Syntech) was used to generate a stimulus at 1-min intervals. Humidified pure air (0.7 L/min) was directed onto the antenna via a 10 mm-diameter glass tube. Sesquiterpenes were obtained from Sigma-Aldrich (St. Louis, MO, USA) and were used as received, without further purification: caryophyllene (≥80% purity), α-farnesene (isomeric mixture, high-purity aroma grade), (+)-longifolene (≥75%), (+)-aromadendrene (96%), α-cedrene (≥95%), and (+)-α-longipinene (≥97%). Liquid trimedlure (≥95%) was obtained from Moscamed program. Compounds were stored at 4 °C in amber vials until use to minimize degradation and isomerization. Four concentrations of the compounds (0.001, 0.01, 0.1, and 1 μg/μL) were tested, with hexane (the solvent) as the control. Filter paper squares (1.5 × 1.5 mm) were impregnated with 10 μL of the treatment, exposed to air for 20 s to allow the solvent to evaporate, and then placed in glass Pasteur pipettes for 20 s before application. New pipettes with treatments were prepared for each antenna. To present a stimulus, the pipette tip containing the piece of filter paper was inserted into a hole at the end of the tube carrying the air stream. The treatment was applied by puffing air through the filter paper at a controlled rate (0.5 L/min). The stimulus duration was 1 s. The continuous flow of air over the preparation ensured that odors were removed from the vicinity. Ten replicates were performed for each concentration of each compound. Antennae from different flies were treated as independent biological replicates, with each preparation corresponding to a distinct individual. EAG assays were conducted using irradiated males provided by the Moscamed program, which routinely supplies sterile flies for experimental purposes. The use of irradiated males ensured biosafety and consistency across assays, and their antennal olfactory responses are physiologically comparable to those of wild-type males, as reported in previous studies.

### 2.5. Statistical Analysis

Data were analyzed using R version 4.3.0 [29]. Tests for homogeneity of variance and normality were applied to the datasets. When necessary, a Box–Cox transformation was applied to ensure the model’s assumptions were met. Docking data were analyzed using one-way analysis of variance (ANOVA) per experiment, with a post hoc Tukey’s HSD test (α = 0.05) implemented in the Agricolae package, version1.3-7 [30]. Binding energy values from the 14 OBPs and 4 ORs were treated as replicates for each compound, with binding energy as the response variable and compound identity as the factor. Docking scores were analyzed separately for OBPs and ORs. Within each protein class, binding energies were treated as pseudo-replicates and averaged to generate descriptive rankings of candidate ligands. ANOVA was applied within each group (OBPs or ORs) to highlight relative differences among compounds, but these values are not intended to represent independent biological replicates. The statistical tests are therefore exploratory and serve only to support the prioritization of ligands for further validation. Importantly, these analyses are exploratory, and *p*-values are reported only to support ranking consistency, not to test hypotheses of statistical independence. EAG responses (in mV) were analyzed using two-way ANOVA to assess the effects of compound identity, concentration, and their interaction. Tukey’s HSD test was used for post hoc comparisons to identify significant differences among treatment levels.

## 3. Results

### 3.1. Odorant-Binding Proteins

**Figure 1:**
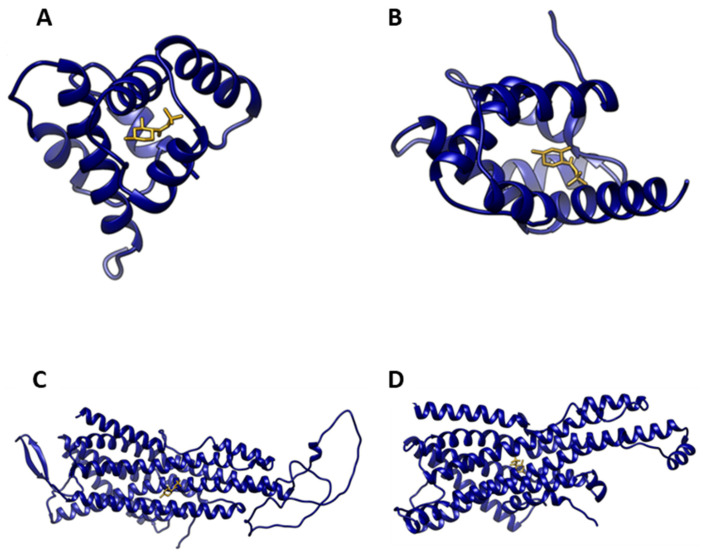
Representative docking poses of trimedlure C with selected medfly OBPs and ORs. Panels show predicted ligand–protein interactions for: (**A**) CcapOBP84a-2, (**B**) CcapOBP99c, (**C**) CcOR83b, and (**D**) CcOR85b. Proteins are represented in navy blue, whereas trimedlure C is represented in goldenrod. Additional docking poses are provided in Supplementary Figure S2.

Trimedlure is a mixture of sixteen stereoisomers of two compounds: t-butyl-4-chloro-2-methylcyclohexanecarboxylate and t-butyl-5-chloro-2-methylcyclohexanecarboxylate. Stereoisomers were designated as *trans* and *cis* according to the substituents at carbons 1 and 2 of the cyclohexane, labeled A, B1, B2, C, V, W, X, and Y, with each letter representing a pair of enantiomers (Supplementary Figure S1) [31]. Representative docking poses illustrate the predicted interactions of trimedlure C with selected OBPs and ORs. These examples highlight typical ligand–protein binding conformations observed in the models (Figure 1; Supplementary Figure S2).

**Figure 2:**
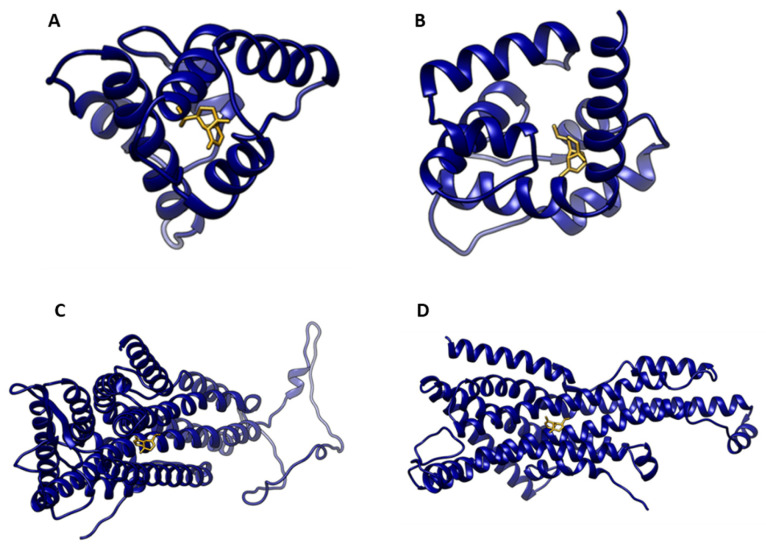
Representative docking poses of α-copaene with selected medfly odorant-binding proteins (OBPs) and odorant receptors (ORs). Panels show predicted ligand–protein interactions for: (**A**) CcapOBP84a-2, (**B**) CcapOBP99c, (**C**) CcOR83b, and (**D**) CcOR85b. Proteins are represented in navy blue, whereas α-copaene is represented in goldenrod. Additional docking poses are provided in Supplementary Figure S3.

Representative docking poses of α-copaene show its predicted interactions with selected OBPs and ORs. These examples complement the binding energy comparisons and provide a visual reference for the ligand–protein associations observed in the models (Figure 2; Supplementary Figure S3).

**Figure 3:**
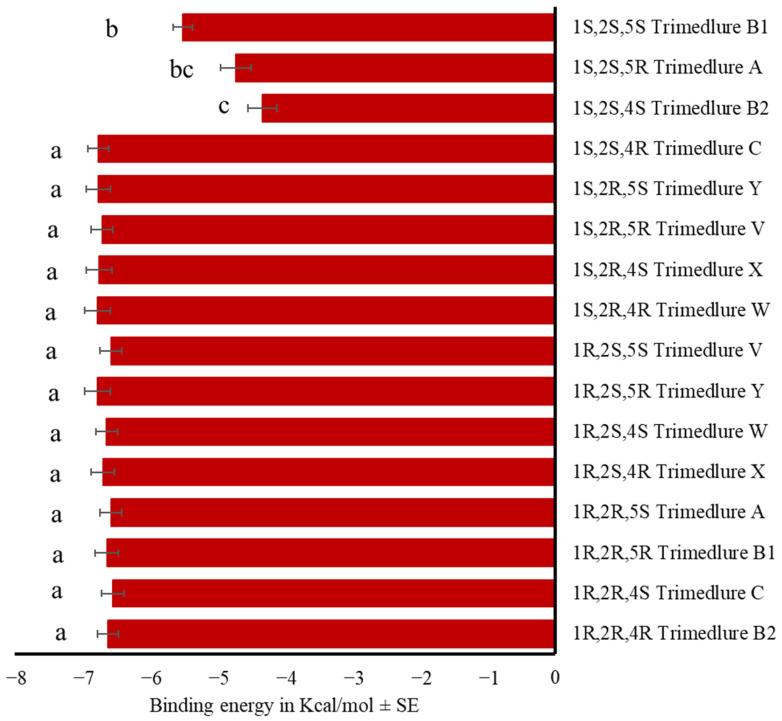
Mean binding energy in Kcal/mol ± SE of the complex OBP-Trimedlure. Stereoisomers are designated A, B1, B2, C, V, W, X, and Y, each letter consisting of a pair of enantiomers. One-way ANOVA (F_15,208_ = 20.59, *p* < 0.001). Different letters indicate significant differences, determined by the Tukey test (α = 0.05).

According to the binding energy of the OBP-trimedlure complex, the isomer 1*S*,2*S*,4*S* trimedlure B2 and the isomer 1*S*,2*S*,5*R* trimedlure A did not bind effectively to the OBPs, followed by the isomer 1*S*,2*S*,5*S* trimedlure B1 (F_15,208_ = 20.59, *p* < 0.001). All other stereoisomers bind similarly to the medfly OBPs (Figure 3, Supplementary Table S3). The isomer 1*S*,2*S*,4*R* trimedlure C is the most active attractant; thus, values above its binding energy (−6.74 ± 0.16 Kcal/mol along the 14 OBPs) were considered potential attractants.

**Figure 4:**
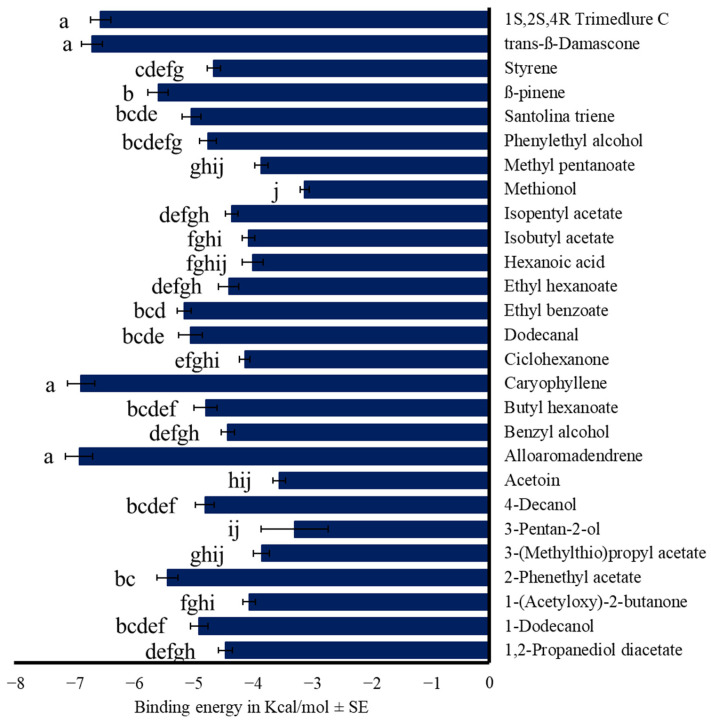
Mean binding energy in Kcal/mol ± SE of the complex OBP-compound. Fermented-food origin compounds compared with 1*S*,2*S*,4*R* trimedlure C. One-way ANOVA (F_27,362_ = 34.16, *p* < 0.001). Different letters denote significant differences, determined by the Tukey test (α = 0.05).

**Figure 5:**
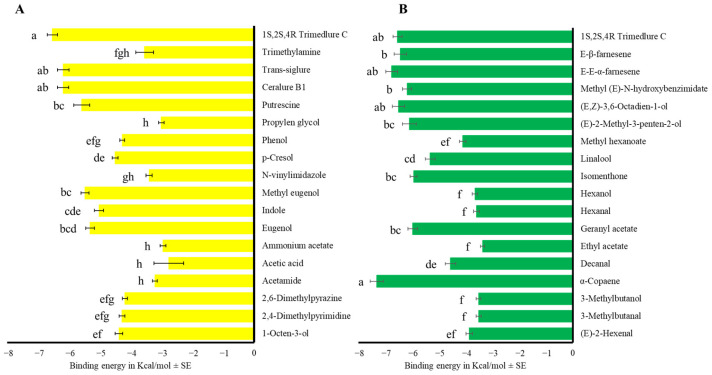
Mean binding energy in Kcal/mol ± SE of the complex OBP-compound compared with 1*S*,2*S*,4*R* trimedlure C. (**A**) Commercial attractants. (**B**) Fruit fly semiochemicals. One-way ANOVA (F_17,237_ = 45.54, *p* < 0.001), and (F_17,234_ = 69.18, *p* < 0.001) respectively. Different letters denote significant differences, determined by the Tukey test (α = 0.05).

We calculated the binding energy values for the 14 OBPs with compounds reported as attractants for the medfly, focusing on essential oil-based terpenoids and fermented-origin compounds (F_27,362_ = 34.16, *p* < 0.001, Figure 4 and Supplementary Table S4), commercial attractants (F_17,237_ = 45.54, *p* < 0.001, Figure 5A and Supplementary Table S4), and fruit fly semiochemicals (F_17,234_ = 69.18, *p* < 0.001, Figure 5B and Supplementary Table S4).

Based on these values, some compounds show binding energies similar to that of trimedlure C. For example, *trans*-Siglure (−6.18 ± 0.18 Kcal/mol) and Ceralure B1 (−6.19 ± 0.18 Kcal/mol), both of which are commercial attractants for medflies (Figure 5A). The pheromone component (*E*,*Z*)-3,6-octadien-1-ol (−6.51 ± 0.23 Kcal/mol), the terpenoid *trans*-β-damascone (−6.69 ± 0.16 Kcal/mol), and the sesquiterpenes α-copaene (−7.32 ± 0.24 Kcal/mol), alloaromadendrene (−6.90 ± 0.23 Kcal/mol), and (*E*-*E*)-α-farnesene (−6.76 ± 0.22 Kcal/mol) (Figure 5B).

**Figure 6:**
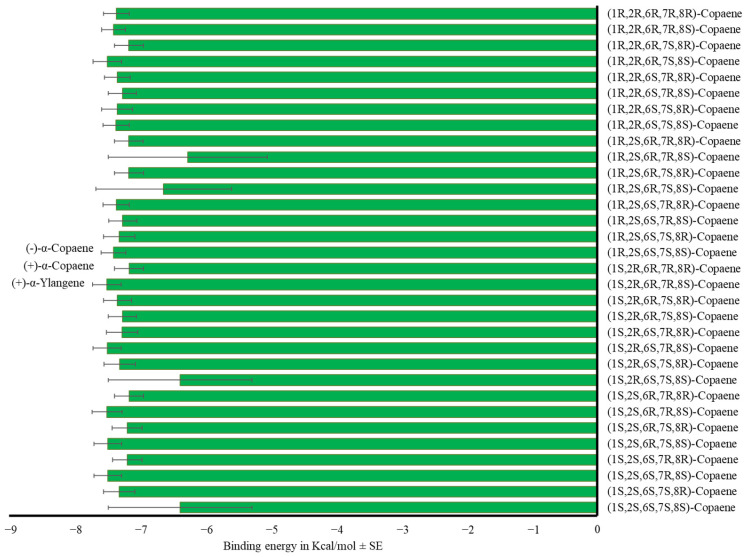
Mean binding energy in Kcal/mol ± SE of the complex OBP-α-copaene using the 32 stereoisomers of the compound. One-way ANOVA (F_31,416_ = 0.59, *p* = 0.96).

We did not find differences in the binding energy of α-copaene isomers (F_31,416_ = 0.59, *p* = 0.96) with the OBPs (Figure 6, Supplementary Table S5).

**Figure 7:**
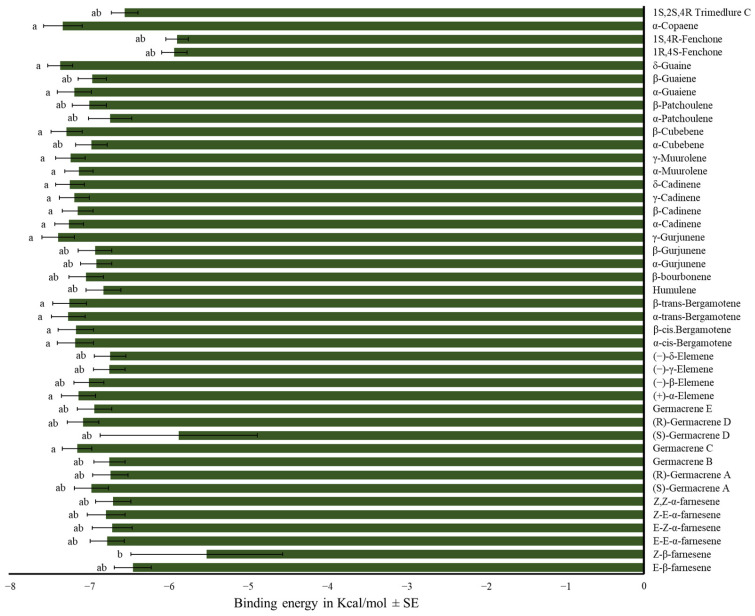
Mean binding energy in Kcal/mol ± SE of the complex OBP-sesquiterpene using selected sesquiterpenes. One-way ANOVA (F_42,559_ = 2.18, *p* < 0.01). Different letters denote significant differences, determined by the Tukey test (α = 0.05).

α-Farnesene isomers showed the same affinity for the medfly’s OBPs. All evaluated sesquiterpenes, except for (*Z*)-β-farnesene, exhibited equal or lower binding energy to OBPs than trimedlure C (F_42,559_ = 2.18, *p* < 0.01, Figure 7, Supplementary Table S6).

### 3.2. Odorant Receptors

**Figure 8:**
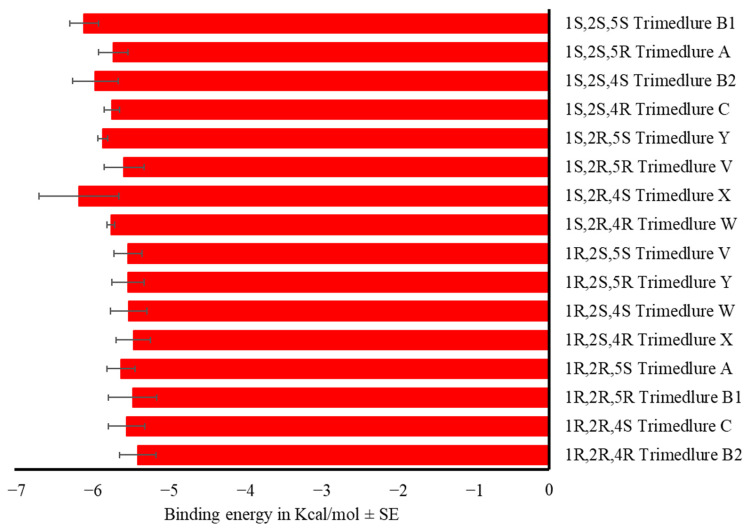
Mean binding energy in Kcal/mol ± SE of the complex OR-trimedlure. Stereoisomers are designated A, B1, B2, C, V, W, X, and Y, each letter consisting of a pair of enantiomers. One-way ANOVA (F_27,36_ = 1.09, *p* = 0.40).

**Figure 9:**
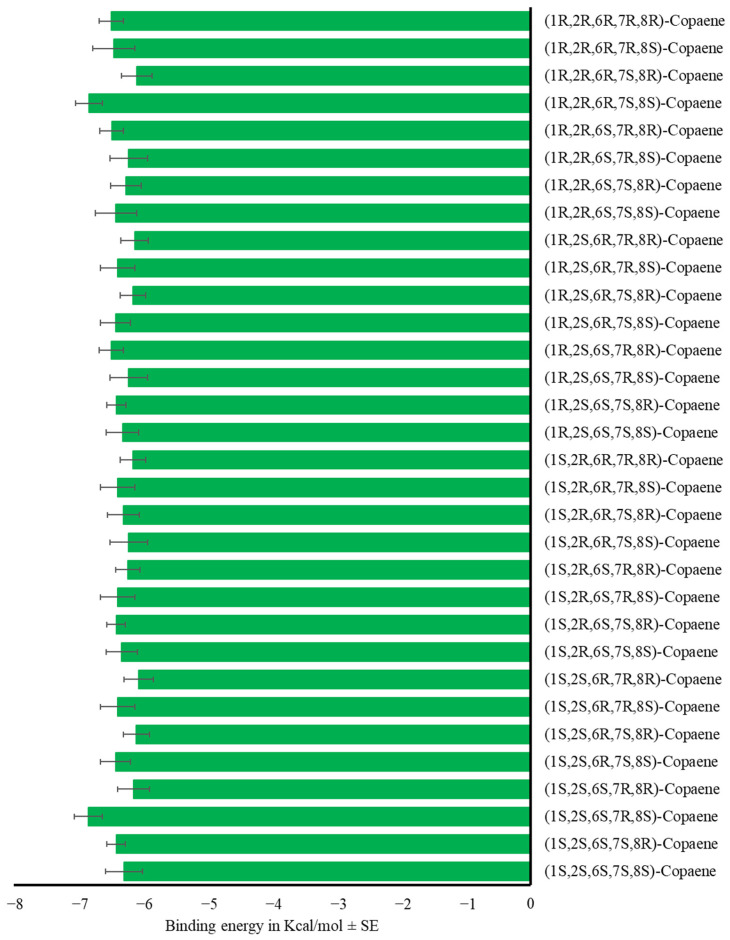
Mean binding energy in Kcal/mol ± SE of the complex OR-α-copaene using the 32 stereoisomers of the compound. One-way ANOVA (F_31,416_ = 0.60, *p* = 0.95).

Trimedlure isomers bind to ORs with the same affinity (F_27,36_ = 1.09, *p* = 0.40, Figure 8, Supplementary Table S7). The same occurs with the α-copaene isomers (F_31,96_ = 0.60, *p* = 0.95, Figure 9, Supplementary Table S8).

**Figure 10:**
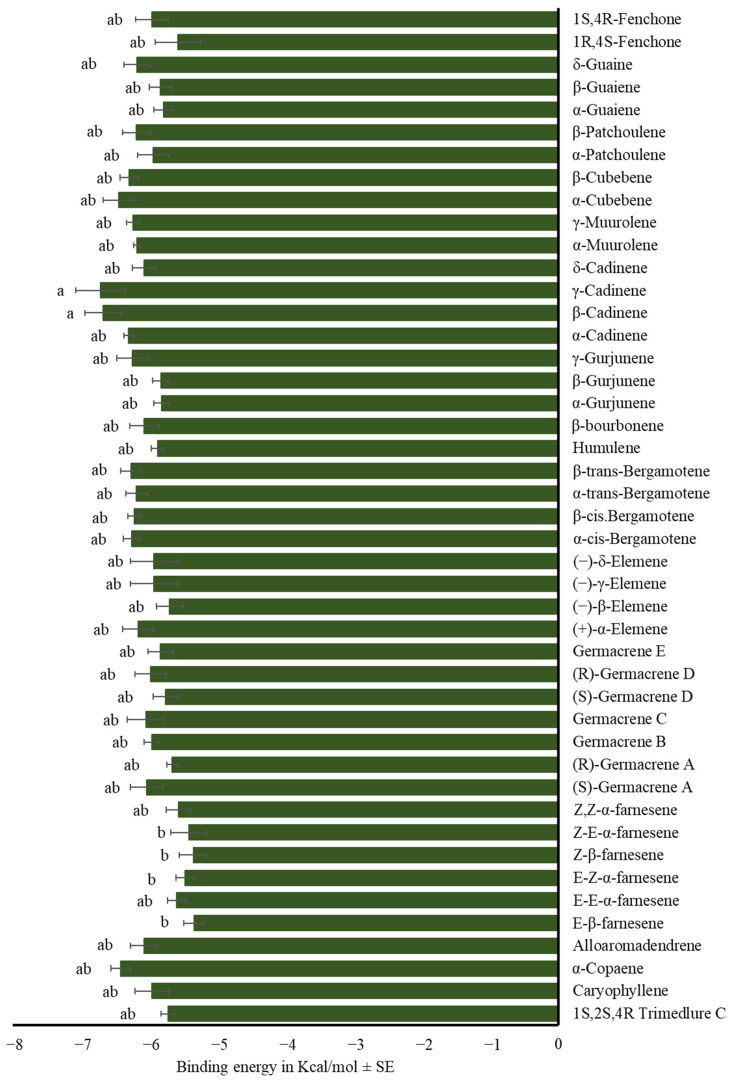
Mean binding energy in Kcal/mol ± SE of the complex OR-sesquiterpene using selected sesquiterpenes. One-way ANOVA (F_44,135_ = 2.56, *p* < 0.001). Different letters denote significant differences, determined by the Tukey test (α = 0.05).

Among other sesquiterpenes, both (*Z*,*E*)- and (*E*,*Z*)-α-farnesene had higher binding energies than trimedlure C. Other tested sesquiterpenes exhibited the same affinity for the medfly ORs as trimedlure C (F_44,135_ = 2.56, *p* < 0.001; Figure 10, Supplementary Table S9).

### 3.3. Electroantennography

**Figure 11:**
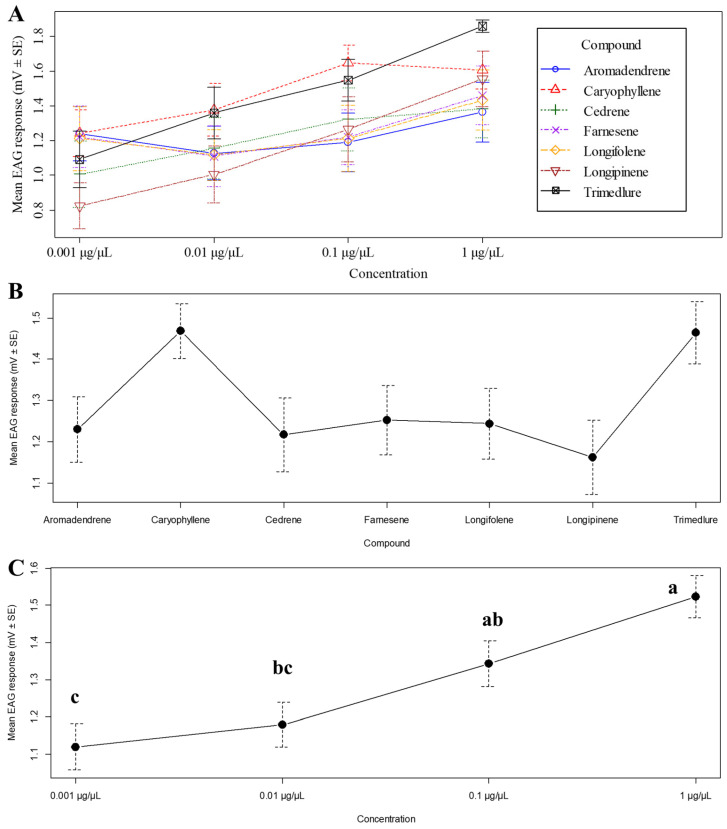
Mean electroantennographic (EAG) responses (±SE) of medfly males. Solvent control values were subtracted from all compound responses to correct for baseline activity. (**A**) Overall mean responses across compounds and concentrations. (**B**) Mean responses by compound (pooled across concentrations). (**C**) Mean responses by concentration (pooled across compounds), different letters above bars indicate significant differences among treatments (Tukey’s HSD test, *p* < 0.05).

EAG responses were significantly affected by concentration (F_3,252_ = 9.10, *p* < 0.001, Figure 11A), indicating a dose-dependent activation of the antennae across the tested compounds. The effect of the compounds was non-significant (F_6,252_ = 3.41, *p* = 0.08, Figure 11B), suggesting a possible trend in differential antennal responses among compounds. No significant interaction was observed between compound and concentration (F_18,252_ = 0.60, *p* = 0.90, Figure 11C, Supplementary Table S10), indicating that the relative response patterns across concentrations were consistent among compounds.

## 4. Discussion

In this study, we conducted molecular docking analyses to assess the interactions between medfly OBPs and ORs with a panel of attractants and semiochemicals, including various stereoisomers. The dataset included fourteen OBPs and four putative ORs. To complement the *in silico* screening, we evaluated the EAG responses of irradiated medfly males to selected sesquiterpenes and trimedlure. The *in silico* method allowed for high-throughput screening of ligand-receptor affinities. Because averaged docking scores dilute target-specific selectivity, they should not be used to draw mechanistic conclusions about OBP/OR-ligand recognition. Docking scores were averaged across multiple OBPs and ORs, so protein-specific patterns of stereoselectivity cannot be determined. It is important to note that docking values were averaged within OBPs and within ORs, and treated as pseudo-replicates for exploratory purposes. The ANOVA results should be interpreted as descriptive comparisons of relative binding affinities rather than inferential tests of protein independence. This approach allowed us to identify compounds with consistently low binding energies across multiple receptors, thereby prioritizing candidates for electrophysiological and behavioral validation. Therefore, the present results do not allow conclusions about selective recognition by individual receptors. The average score is used only as a ranking tool to prioritize candidates for experimental testing.

In molecular docking, lower binding free energy signifies a more stable ligand–receptor complex, which often correlates with higher biological activity. As a result, compounds with the lowest predicted binding energies were considered promising candidates for behavioral activity [32]. Low docking scores should be interpreted only as reduced predicted affinity, not as evidence of the absence of binding. Experimental validation is required to determine whether these compounds are truly inactive. Trimedlure isomers were included as reference compounds in the docking analysis, given their established role as male attractants in medfly management programs [33]. *Trans*-isomers are the most common compounds in commercial formulations and are reported to be more effective attractants than *cis*-isomers. However, although previous field studies reported the ranking C > A > Y > B1 > V > X > W > B2, our docking scores only partially match this pattern, which highlights the complexity of stereoselective interactions and the limitations of docking alone to predict field attractiveness [34,35]. However, field-recapture experiments were conducted using pairs of enantiomers; studies with individual enantiomers have used the *trans*-isomers [34]. The enantiomer 1*S*,2*S*,4*R* of trimedlure C is the most attractive for the medfly, followed by the enantiomer 1*R*,2*R*,5*S* of trimedlure A. Other *trans* stereoisomers are 5–10 times less attractive than these two isomers [31]. Meanwhile, studies on *cis*-isomers using individual enantiomers are lacking. The most attractive isomers, trimedlure C (1*R*,2*R*,4*S* and 1*S*,2*S*,4*R*) and A (1*R*,2*R*,5*S*), exhibited the low binding energies across the 14 OBPs, indicating strong protein-ligand interactions. Conversely, less attractive isomers such as 1*S*,2*S*,5*S*-B1, 1*S*,2*S*,4*S*-B2 and 1*S*,2*S*,5*R*-A showed significantly weaker binding. This dependence on stereochemistry aligns with the compound’s attractiveness in the field and with the antennal responses of male and female medflies [31,33,34,36]. All trimedlure stereoisomers showed comparable binding affinities across the four modeled ORs, suggesting minimal stereoselectivity at the receptor level. This result contrasts with the moderate stereoselectivity observed in OBPs, which may serve as a preliminary filter before odorants reach the ORs. However, further Single *Sensillum* Recordings (SSR) and behavioral assays with individual stereoisomers are needed to confirm this hypothesis and assess whether specific ORNs exhibit more nuanced enantioselectivity. Additionally, future research should address the interactions between OBPs and ORs. Such studies would provide deeper insight into the molecular mechanisms of olfactory recognition in *C. capitata*, an area where the current literature remains limited.

Compounds from essential oils have potential attraction effects on the medfly [37]. Oils such as ginger root, orange, manuka, angelica, cubeb, and tea tree are potentially attractive due to their potential to enhance mating success [38,39]. Additionally, fermented-food compounds [40] and semiochemicals from other fruit flies [18,41] have been reported for controlling the medfly. *Trans*-siglure was the first synthetic attractant used to control the medfly; this was followed by the synthesis of many structural analogs, such as trimedlure and ceralure B1 (the iodinated analog of trimedlure), which were demonstrated to be better attractants for the medfly [34]. Ceralure B has been reported to be a better attractant for medflies than trimedlure. However, the synthesis costs appear to be higher for ceralure B [42]. In the present study, docking and EAG assays confirmed that sesquiterpenes, such as α-copaene and (*E*,*E*)-α-farnesene, are detectable by the medfly olfactory system. These findings highlight the potential of sesquiterpenes as candidate attractants. In comparison, other terpenoids such as *trans*-β-damascone, which occurs in fruit fermentation, and (*E*,*Z*)-3,6-octadien-1-ol, produced by calling males of *Anastrepha obliqua* [43], have not yet been evaluated as lures for *C. capitata.* While not part of the present study, these compounds represent promising candidates for future bioassays of medfly attraction.

It is well known that some sesquiterpenes are potential attractants for the medfly [44,45]. The sesquiterpene α-copaene is present in the medfly hosts and serves as a primary male attractant and cue for lek formation. It is linked to male mating functions and has been shown to increase mating frequency by altering the male mating pheromone [39]. There is no association between α-copaene levels and the male response; this may be due to differences in isomeric structures among medfly host plants [46]. The three-dimensional structure of α-copaene resembles that of ceralure B in the binding site of the OBPs [17]. Analogs of α-copaene have been evaluated as attractants of the medfly [45], indicating that (+)-α-copaene is the most active isomer. Still, the authors did not consider all the possible isomers. The docking experiments, performed in this study, revealed that all α-copaene stereoisomers exhibited similar binding energies across the 14 OBPs and 4 ORs, indicating low stereoselectivity. As heteromeric ligand-gated ion channels located in the antennae and palps, the ORs play a central role in olfaction. They may tolerate substantial structural variation in ligands, an insight that could be valuable for the rational design of new attractants [47]. These suggest that the stereochemical configuration of these compounds does not critically influence the activation of the corresponding heteromeric ligand-gated ion channels in insect olfactory neurons. However, enantiomeric discrimination in insect olfaction and chemical ecology remains unclear [16]. The apparent lack of stereoselectivity observed in our results suggests that the ligand-binding domains of these ORs may accommodate a variety of conformational isomers, recognizing shared structural motifs conserved across stereoisomers. Docking values were averaged within OBPs and within ORs and treated as pseudo-replicates for exploratory purposes. Four representative proteins were selected to illustrate typical ligand–protein interactions: CcapOBP84a-2 and CcapOBP99c with consistently strong affinities, and CcOR83b and CcOR85b with distinct binding profiles. The figures illustrate α-copaene and trimedlure C within binding sites, thereby contextualizing the averaged docking results.

The binding energies of the stereoisomers of α-copaene and α-farnesene, across the 14 OBPs and 4 ORs, appear to be independent of chirality, suggesting that they are potential ligands. Furthermore, a natural oil mixture enriched with α-copaene was as effective as, or even more effective than, fresh liquid trimedlure in capturing medfly males for several weeks [8,48]. (*E*,*E*)-α-Farnesene is part of the medfly male sex pheromone, along with compounds like geranyl acetate and linalool [12]. Additionally, medfly immature and mature virgin females responded to (*Z*,*E*)-α-farnesene in EAG experiments [49], and it attracts both sexes [10]. These could indicate the potential for designing lures that capture both sexes. Compounds such as bergamotenes had a lower binding energy than trimedlure C. However, these compounds were assessed in recapture experiments, which yielded no recaptures [45]. This suggests that these compounds are effective repellents against the medfly, but bioassays are needed to confirm this. Bioassay evaluation of individual sesquiterpenes or their mixtures holds promise for identifying effective attractants for the medfly. However, one significant problem with using sesquiterpenes as lures is the high cost of obtaining them (by synthesis or extraction) and purifying them [50]. But if the OBPs and ORs did not discriminate stereoisomerism, those compounds could be used as diastereomeric or enantiomeric mixtures. However, future studies employing SSR or heterologous expression assays will be necessary to validate these findings and assess whether ORNs exhibit more refined enantioselectivity.

According to the EAG recordings, the antennal responses elicited by the evaluated sesquiterpenes were comparable to those induced by trimedlure and were dose-dependent. This suggests that these natural compounds can activate the peripheral olfactory system of medfly males to a similar extent as the synthetic benchmark. Because compound-specific differences were statistically weak, we interpret these findings as evidence of antennal detectability rather than differential potency. Behavioral assays will be necessary to determine whether these compounds differ in attractiveness under field conditions. Notably, compounds such as α-copaene and α-farnesene, which are components of the male-produced pheromone blend, elicited strong responses, underscoring their ecological relevance. It would be valuable to assess the effectiveness of these sesquiterpenes when applied in the same amounts and formulations as those used in commercial trimedlure plugs. Additionally, testing enantiomerically pure compounds or isomer-enriched mixtures could help determine whether the antennal responses are influenced by chirality at the physiological level, complementing the docking results.

Functional plasticity may confer an adaptive advantage, enabling insects to reliably detect ecologically relevant odorants despite natural variation in chemical composition. Such receptor flexibility could enhance the robustness of chemosensory perception in complex olfactory landscapes, aiding vital behaviors such as host location, mate finding, and oviposition site selection [51]. That supports the hypothesis that sesquiterpenes and trimedlure target the same ORs, reinforcing the idea of receptor flexibility in detecting structurally diverse ligands [48]. However, further studies are needed to evaluate ORN responses in SSR and bioassays, as well as to determine the importance of chirality throughout the process [21]. From an applied perspective, attractant formulations could use commercially available mixtures of sesquiterpenes, thereby avoiding the cost of purifying individual compounds. This would simplify lure production and potentially reduce the cost of pest-monitoring or control programs. In this regard, screening the wide diversity of sesquiterpenes and sesquiterpenoids could lead to the discovery of attractants for both medfly males and females. Nevertheless, the use of mixed sesquiterpenes could reduce production and purification costs; practical application will require careful consideration of formulation and release kinetics. Factors such as oxidation stability, controlled release rates, and field longevity are critical constraints that must be addressed in future studies. Optimizing delivery systems will be essential to translating these promising candidates into effective, durable attractants for integrated pest management programs. Additionally, efforts are needed to improve the efficiency of their synthesis [50,52]. Docking analyses and EAG recordings confirm that sesquiterpenes are detectable by the medfly olfactory system and elicit dose-dependent antennal activation. However, while these compounds represent promising candidates for further evaluation, their potential to substitute or complement trimedlure as attractants can only be established through behavioral assays under field conditions.

## 5. Conclusions and Future Directions

This study provides valuable insights into potential interactions in the olfactory detection mechanisms of the medfly, based on docking analyses of 14 OBPs, 4 ORs, and over 100 known attractant compounds, including stereoisomers of trimedlure and various sesquiterpenes. This work demonstrates the usefulness of a combined in silico and electrophysiological workflow for identifying potential attractants. Although other promising compounds identified through docking (e.g., *trans*-β-damascone and (*E*,*Z*)-3,6-octadien-1-ol) were not included in the initial EAG assays due to limited availability, they remain strong candidates for future testing.

Several sesquiterpenes displayed binding energies equal to or lower than that of trimedlure C, underscoring their potential as alternative semiochemicals for managing the medfly. Importantly, all α-copaene and trimedlure isomers exhibited binding energies to ORs that were not statistically distinguishable, indicating minimal stereoselectivity at the receptor level. This receptor plasticity likely reflects an adaptive mechanism that allows the medfly to recognize ecologically diverse volatiles despite structural variability. Our findings emphasize the value of integrating molecular docking with electrophysiological and behavioral studies to identify and prioritize novel attractants. The broad chemical-recognition capacity of medfly olfactory proteins offers opportunities to develop next-generation lures that target both sexes, based on sesquiterpene scaffolds. From an applied standpoint, this lack of stereochemical discrimination supports the use of racemic or unrefined isomeric mixtures in attractant formulations, thereby substantially reducing production and purification costs. The exclusive use of irradiated males in this study reflects the operational context of sterile insect release programs and provides a preliminary approach to identify compounds detectable by males deployed in pest management. While antennal responses of irradiated males are considered physiologically comparable to those of wild-type males, future studies should include females and non-irradiated males to evaluate potential differences in olfactory perception and to broaden the applicability of candidate attractants. Additionally, future work should include SSR to assess ORN specificity, bioassays to identify optimal release devices, and efforts to improve sourcing, synthesis, and deployment of these compounds for sustainable IPM programs.

## Data Availability

The original contributions presented in this study are included in the article/Supplementary Materials. Further inquiries can be directed to the corresponding author.

## Supplementary Materials

The following supporting information can be downloaded at: https://www.mdpi.com/article/10.3390/insects17030251/s1, Figure S1: Stereoisomers of t-butyl-4-chloro-2-methylcyclohexanecarboxylate and t-butyl-5-chloro-2-methylcyclohexanecarboxylate, named *trans* and *cis* according to substituents in carbon 1 and 2 of the cyclohexane, are designated A, B1, B2, C, V, W, X, and Y, each letter consisting of a pair of enantiomers; Figure S2: Representative molecular docking poses of trimedlure C with medfly odorant-binding proteins (OBPs) and odorant receptors (ORs). Panels show the predicted ligand–protein interactions for: (A) CcapOBP19a, (B) CcapOBP19b, (C) CcapOBP19d-1, (D) CcapOBP28a, (E) CcapOBP44a, (F) CcapOBP49a, (G) CcapOBP56d, (H) CcapOBP56h, (I) CcapOBP69a, (J) CcapOBP83a, (K) CcapOBP84a-2, (L) CcapOBP99a, (M) CcapOBP99c, (N) CcapOBP99d, (O) CcOR7a, (P) CcOR59b, (Q) CcOR83b, and (R) CcOR85b; Figure S3: Representative molecular docking poses of α copaene with medfly odorant binding proteins (OBPs) and odorant receptors (ORs). Panels show the predicted ligand–protein interactions for: (A) CcapOBP19a, (B) CcapOBP19b, (C) CcapOBP19d 1, (D) CcapOBP28a, (E) CcapOBP44a, (F) CcapOBP49a, (G) CcapOBP56d, (H) CcapOBP56h, (I) CcapOBP69a, (J) CcapOBP83a, (K) CcapOBP84a 2, (L) CcapOBP99a, (M) CcapOBP99c, (N) CcapOBP99d, (O) CcOR7a, (P) CcOR59b, (Q) CcOR83b, and (R) CcOR85b; Table S1: Semiochemicals, attractants, and stereoisomers tested in this study, including formula, molecular weight, CAS number, and chemical class; Table S2: Structural model quality assessment; Table S3: Values of the binding energy (in Kcal/mol) of the sixteen Trimedlure isomers with the fourteen OBPs; Table S4: Values of the binding energy (in Kcal/mol) of the fourteen OBPs with commercial attractants, fermented-origin compounds, and semiochemicals; Table S5: Values of the binding energy (in Kcal/mol) of the fourteen OBPs with stereoisomers of α-copaene; Table S6: Values of the binding energy (in Kcal/mol) of the fourteen OBPs with stereoisomers of some sesquiterpenes; Table S7: Values of the binding energy (in Kcal/mol) of the sixteen Trimedlure isomers with the four ORs of *C. capitata*; Table S8: Values of the binding energy (in Kcal/mol) of the four ORs with stereoisomers of α-copaene; Table S9: Values of the binding energy (in Kcal/mol) of the four ORs with stereoisomers of some sesquiterpenes; Table S10: Values the antennal response (in mV) of the medfly.

## Abbreviations

The following abbreviations are used in this manuscript:

OBP	Odorant Binding Protein
OR	Odorant Receptor
EAG	Electroantennography
IPM	Integrated Pest Management
