# Supplementary material for: Integrating Molecular Docking and Electrophysiology Reveals Sesquiterpenes as Candidate Attractants for Ceratitis capitata Wiedemann (Diptera: Tephritidae)
Source: Insects. 2026 Feb 27;17(3):251. doi: 10.3390/insects17030251 (PMC13026091; doi:10.3390/insects17030251)
Supplement: Supplementary file 1 [file insects-17-00251-s001.zip › insects-4163785-supplementary.pdf]

Integrating molecular docking and electrophysiology reveals sesquiterpenes as candidate attractants for *Ceratitis capitata* Wiedemann (Diptera: Tephritidae).

Daniela Ordaz-Pérez[1] Julio C. Rojas[2], & David Alavez-Rosas [2,3*]

[1] Laboratorio de Taxonomía, Programa Operativo de Moscas, Planta MOSCAFRUT, Metapa de Domínguez 30826, Chiapas, México; daniela.ordaz.i@senasica.gob.mx

[2] Grupo de Manejo de Plagas y Vectores de Enfermedades, Departamento de Ecología de Artrópodos y Manejo de Plagas, El Colegio de la Frontera Sur (ECOSUR), Carretera Antiguo Aeropuerto Km. 2.5, Tapachula 30700, Chiapas, México

[3] Grupo de Ecología Química, Departamento de Ecología de Artrópodos y Manejo de Plagas, El Colegio de la Frontera Sur (ECOSUR), Carretera Antiguo Aeropuerto Km. 2.5, Tapachula 30700, Chiapas, México; jrojas@ecosur.mx

[4] Grupo Colegiado de Investigación en Educación en Ciencias Químico Fármaco Biológicas y Salud Ambiental, Facultad de Ciencias Químicas, Benemérita Universidad Autónoma de Chiapas, Carretera a Puerto Madero Km. 1.5, Tapachula 30792, Chiapas, México

***** Correspondence: daalavez@ecosur.edu.mx

Supporting information

Supplementary Figure S1. Stereoisomers of t-butyl-4-chloro-2-methylcyclohexanecarboxylate and t-butyl-5-chloro-2-methylcyclohexanecarboxylate, named *trans* and *cis* according to substituents in carbon 1 and 2 of the cyclohexane, are designated A, B1, B2, C, V, W, X, and Y, each letter consisting of a pair of enantiomers.

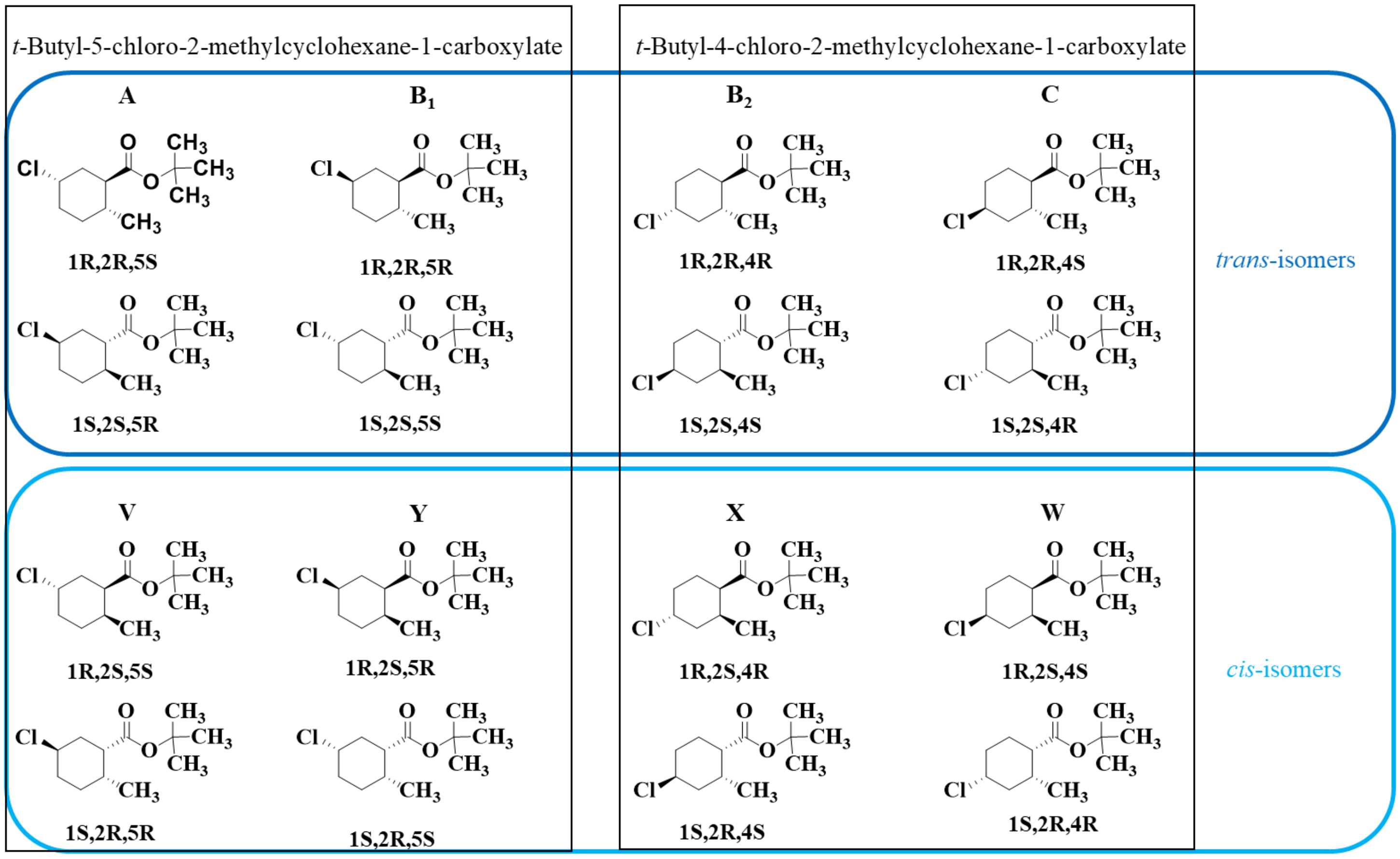

Supplementary Figure S2. Representative molecular docking poses of trimedlure C with medfly odorant-binding proteins (OBPs) and odorant receptors (ORs). Panels show the predicted ligand–protein interactions for: A) CcapOBP19a, B) CcapOBP19b, C) CcapOBP19d-1, D) CcapOBP28a, E) CcapOBP44a, F) CcapOBP49a, G) CcapOBP56d, H) CcapOBP56h, I) CcapOBP69a, J) CcapOBP83a, K) CcapOBP84a-2, L) CcapOBP99a, M) CcapOBP99c, N) CcapOBP99d, O) CcOR7a, P) CcOR59b, Q) CcOR83b, and R) CcOR85b.

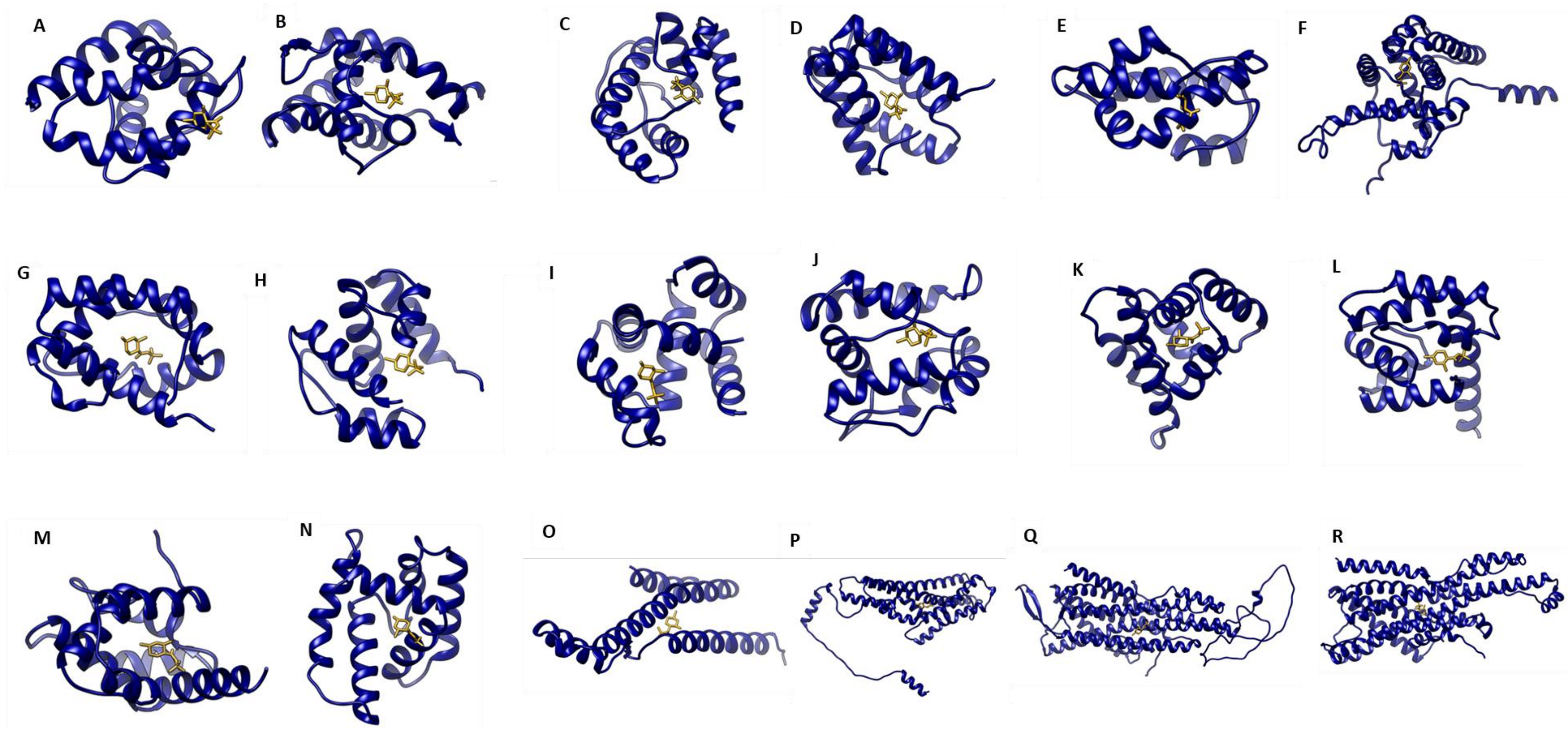

Supplementary Figure S3. Representative molecular docking poses of α-copaene with medfly odorant-binding proteins (OBPs) and odorant receptors (ORs). Panels show the predicted ligand–protein interactions for: A) CcapOBP19a, B) CcapOBP19b, C) CcapOBP19d-1, D) CcapOBP28a, E) CcapOBP44a, F) CcapOBP49a, G) CcapOBP56d, H) CcapOBP56h, I) CcapOBP69a, J) CcapOBP83a, K) CcapOBP84a-2, L) CcapOBP99a, M) CcapOBP99c, N) CcapOBP99d, O) CcOR7a, P) CcOR59b, Q) CcOR83b, and R) CcOR85b.

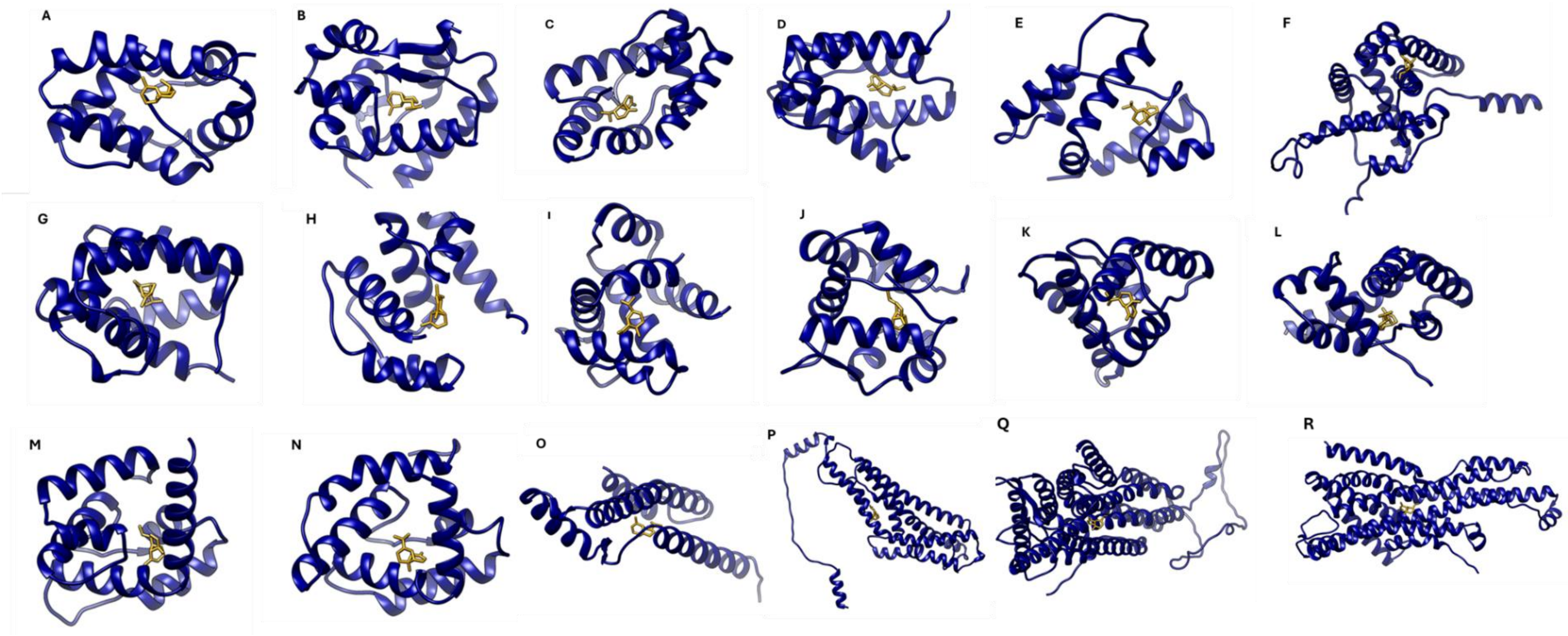

Supplementary Table S1. Semiochemicals, attractants, and stereoisomers tested in this study, including formula, molecular weight, CAS number, and chemical class.

Entry	Compound name	Molecular formula	Molecular weight (g/mol)	CAS number	Chemical class
**Attractants**					
1	1-Octen-3-ol	C8H16O	128.21	3391-86-4	Alcohol
2	2,4-Dimethylpyrimidine	C7H9N	107.15	108-47-4	Pyridine derivative
3	2,6-Dimethylpyrazine	C8H11N	121.18	87-62-7	Aromatic amine
4	Acetamide	C2H5NO	59.07	60-35-5	Amide
5	Acetic acid	C2H4O2	60.05	64-19-7	Carboxylic acid
6	Ammonium acetate	C2H7NO2	77.08	631-61-8	Salt (buffer)
7	Eugenol	C10H12O2	164.2	97-53-0	Phenylpropanoid
8	Indole	C8H7N	117.15	120-72-9	Aromatic heterocycle
9	Methyl eugenol	C11H14O2	178.23	93-15-2	Phenylpropanoid ester
10	N-vinylimidazole	C5H6N2	94.11	1072-63-5	Aromatic heterocycle
11	p-Cresol	C_7H_8O	108.13	06-44-5	Aromatic alcohol
12	Phenol	C6H6O	94.11	108-95-2	Aromatic alcohol
13	Propylen glycol	C3H8O2	76.09	57-55-6	Polyol
14	Putrescine	C4H12N2	88.15	110-60-1	Diamine
15	Trimethylamine	C3H9N	59.11	75-50-3	Amine
**Fermented-origin compounds**					
16	1,2-Propanediol, diacetate	C7H12O4	160.17	623-84-7	Ester
17	1-Dodecanol	C12H26O	186.33	112-53-8	Alcohol
18	2-Butanone, 1-(acetyloxy)-	C6H10O3	130.14	600-22-6	Ester
19	2-Phenethyl acetate	C10H12O2	164.2	103-45-7	Ester
20	3-(Methylthio)propyl acetate	C6H12O2S	148.23	16630-55-0	Ester
21	3-Penten-2-ol	C5H10O	86.13	1569-50-2	Alcohol
22	4-Decanol	C10H22O	158.28	2051-31-2	Alcohol
23	Acetoin	C4H8O2	88.11	513-86-0	Hydroxiketone
24	Alloaromadendrene	C15H24	204.35	25246-27-9	Sesquiterpene
25	Benzyl alcohol	C7H8O	108.14	100-51-6	Aromatic alcohol
26	Butyl hexanoate	C13H18O2	206.28	6789-88-4	Ester
27	Caryophyllene	C15H24	204.35	87-44-5	Sesquiterpene
28	Ciclohexanone	C6H10O	98.14	108-94-1	Ketone
29	Dodecanal	C12H24O	184.32	112-54-9	Aldehyde
30	Ethyl benzoate	C9H10O2	150.17	93-89-0	Ester

31	Ethyl hexanoate	C8H16O2	144.21	123-66-0	Ester
32	Hexanoic acid	C6H12O2	116.16	142-62-1	Carboxylic acid
33	Isobutyl acetate	C6H12O2	116.16	110-19-0	Ester
34	Isopentyl acetate	C7H14O2	130.18	123-92-2	Ester
35	Methionol	$C_4H_{10}OS$	1006.18	505-10-2	Alcohol
36	Methyl hexanoate	C7H14O2	130.19	106-70-7	Ester
37	Methyl pentanoate	C6H12O2	116.16	624-24-8	Ester
38	Phenylethyl alcohol	C8H10O	122.16	60-12-8	Aromatic alcohol
39	Santolina triene	C10H16	136.23	2153-66-4	Terpene
40	ß-pinene	C10H16	136.23	18172-67-3	Terpene
41	Styrene	C8H8	104.15	100-42-5	Aromatic
42	trans-ß-Damascone	C13H20O	192.3	23726-91-2	Ketone
**Semiochemicals**					
43	(E)-2-hexenal	C6H10O	98.15	6728-26-3	Aldehyde
44	3-Methylbutanal	C5H10O	86.13	590-86-3	Aldehyde
45	3-Methylbutanol	C5H12O	88.15	123-51-3	Alcohol
46	Ceralure B1	C10H17IO2	296.14		Ester
47	Decanal	C10H20O	156.26	112-31-2	Aldehyde
48	Ethyl acetate	C4H8O2	88.11	141-78-6	Ester
49	Geranyl acetate	C12H20O2	196.29	105-87-3	Monoterpene ester
50	Hexanal	C6H12O	100.16	66-25-1	Aldehyde
51	Hexanol	C6H14O	102.17	111-27-3	Alcohol
52	Isomenthone	C10H18O	154.25	491-07-6	Monoterpene ketone
53	Linalool	C10H18O	154.25	78-70-6	Monoterpene alcohol
54	Methyl hexanoate	C7H14O2	130.19	106-70-7	Ester
55	Trans-siglure	C12H20O2	196.29	2425-20-9	Ester
56	(E)-2-Methyl-3-penten-2-ol	C6H12O	100.16		Enol
57	(E,Z)-3,6-octadien-1-ol	C8H14O	126.2		Dienol
58	methyl (E)-N-hydroxybenzimidate	C11H23NO2	201.31		Imidate derivative
**Sesquiterpenes**					
59	E-β-farnesene	C15H24	204.35	18794-84-8	Sesquiterpene
60	Z-β-farnesene	C15H24	204.35	28973-97-9	Sesquiterpene
61	E-E-α-farnesene	C15H24	204.35	502-60-3	Sesquiterpene
62	E-Z-α-farnesene	C15H24	204.35	26560-14-5	Sesquiterpene
63	Z-E-α-farnesene	C15H24	204.35	502-61-4	Sesquiterpene
64	Z,Z-α-farnesene	C15H24	204.35	54397-85-2	Sesquiterpene
65	(S)-Germacrene A	C15H24	204.35	23986-74-5	Sesquiterpene
66	(R)-Germacrene A	C15H24	204.35	1686-67-5	Sesquiterpene
67	Germacrene B	C15H24	204.35	15423-57-1	Sesquiterpene

68	Germacrene C	C15H24	204.35	56957-61-0	Sesquiterpene
69	(S)-Germacrene D	C15H24	204.35	23986-74-5	Sesquiterpene
70	(R)-Germacrene D	C15H24	204.35	23986-74-5	Sesquiterpene
71	Germacrene E	C15H24	204.35	56957-61-0	Sesquiterpene
72	(+)-α-Elemene	C15H24	204.35	515-13-9	Sesquiterpene
73	(−)-β-Elemene	C15H24	204.35	515-13-9	Sesquiterpene
74	(−)-γ-Elemene	C15H24	204.35		Sesquiterpene
75	(−)-δ-Elemene	C15H24	204.35		Sesquiterpene
76	α-cis-Bergamotene	C15H24	204.35	17627-29-1	Sesquiterpene
77	β-cis.Bergamotene	C15H24	204.35	17627-30-4	Sesquiterpene
78	α-trans-Bergamotene	C15H24	204.35		Sesquiterpene
79	β-trans-Bergamotene	C15H24	204.35	17627-31-5	Sesquiterpene
80	Humulene	C15H24	204.35	6753-98-6	Sesquiterpene
81	β-bourbonene	C15H24	204.35		Sesquiterpene
82	α-Gurjunene	C15H24	204.35	17334-56-4	Sesquiterpene
83	β-Gurjunene	C15H24	204.35	17334-55-3	Sesquiterpene
84	γ-Gurjunene	C15H24	204.35	17334-57-5	Sesquiterpene
85	α-Cadinene	C15H24	204.35	29371-13-5	Sesquiterpene
86	β-Cadinene	C15H24	204.35	29371-14-6	Sesquiterpene
87	γ-Cadinene	C15H24	204.35	29371-15-7	Sesquiterpene
88	δ-Cadinene	C15H24	204.35	29371-16-8	Sesquiterpene
89	α-Muurolene	C15H24	204.35	17627-24-6	Sesquiterpene
90	γ-Muurolene	C15H24	204.35		Sesquiterpene
91	α-Cubebene	C15H24	204.35	17699-14-8	Sesquiterpene
92	β-Cubebene	C15H24	204.35	13744-15-5	Sesquiterpene
93	α-Patchoulene	C15H24	204.35	514-51-4	Sesquiterpene
94	β-Patchoulene	C15H24	204.35	514-51-4	Sesquiterpene
95	α-Guaiene	C15H24	204.35	3691-11-0	Sesquiterpene
96	β-Guaiene	C15H24	204.35		Sesquiterpene
97	δ-Guaine	C15H24	204.35	3691-13-2	Sesquiterpene
98	1R,4S-Fenchone	C10H16O	152.23	7787-20-4	Monoterpene ketone
99	1S,4R-Fenchone	C10H16O	152.23	7787-20-4	Monoterpene ketone
**Trimedlure isomers**					
100	1R,2R,4S Trimedlure C	C13H20O2	208.3	12002-53-8	Ester (synthetic lure)
101	1R,2R,4R Trimedlure B2	C13H20O2	208.3	12002-53-8	Ester (synthetic lure)
102	1R,2R,4S Trimedlure C	C13H20O2	208.3	12002-53-8	Ester (synthetic lure)
103	1R,2R,5R Trimedlure B1	C13H20O2	208.3	12002-53-8	Ester (synthetic lure)
104	1R,2R,5S Trimedlure A	C13H20O2	208.3	12002-53-8	Ester (synthetic lure)
105	1R,2S,4R Trimedlure X	C13H20O2	208.3	12002-53-8	Ester (synthetic lure)
106	1R,2S,4S Trimedlure W	C13H20O2	208.3	12002-53-8	Ester (synthetic lure)

107	1R,2S,5R Trimedlure Y	C13H20O2	208.3	12002-53-8	Ester (synthetic lure)
108	1R,2S,5S Trimedlure V	C13H20O2	208.3	12002-53-8	Ester (synthetic lure)
109	1S,2R,4R Trimedlure W	C13H20O2	208.3	12002-53-8	Ester (synthetic lure)
110	1S,2R,4S Trimedlure X	C13H20O2	208.3	12002-53-8	Ester (synthetic lure)
111	1S,2R,5R Trimedlure V	C13H20O2	208.3	12002-53-8	Ester (synthetic lure)
112	1S,2R,5S Trimedlure Y	C13H20O2	208.3	12002-53-8	Ester (synthetic lure)
113	1S,2S,4R Trimedlure C	C13H20O2	208.3	12002-53-8	Ester (synthetic lure)
114	1S,2S,4S Trimedlure B2	C13H20O2	208.3	12002-53-8	Ester (synthetic lure)
115	1S,2S,5R Trimedlure A	C13H20O2	208.3	12002-53-8	Ester (synthetic lure)
116	1S,2S,5S Trimedlure B1	C13H20O2	208.3	12002-53-8	Ester (synthetic lure)
**Copaene isomers**					
117	(1S,2S,6S,7S,8S)-Copaene	C15H24	204.35		Sesquiterpene
118	(1S,2S,6S,7S,8R)-Copaene	C15H24	204.35		Sesquiterpene
119	(1S,2S,6S,7R,8S)-Copaene	C15H24	204.35		Sesquiterpene
120	(1S,2S,6S,7R,8R)-Copaene	C15H24	204.35		Sesquiterpene
121	(1S,2S,6R,7S,8S)-Copaene	C15H24	204.35		Sesquiterpene
122	(1S,2S,6R,7S,8R)-Copaene	C15H24	204.35		Sesquiterpene
123	(1S,2S,6R,7R,8S)-Copaene	C15H24	204.35		Sesquiterpene
124	(1S,2S,6R,7R,8R)-Copaene	C15H24	204.35		Sesquiterpene
125	(1S,2R,6S,7S,8S)-Copaene	C15H24	204.35		Sesquiterpene
126	(1S,2R,6S,7S,8R)-Copaene	C15H24	204.35		Sesquiterpene
127	(1S,2R,6S,7R,8S)-Copaene	C15H24	204.35		Sesquiterpene
128	(1S,2R,6S,7R,8R)-Copaene	C15H24	204.35		Sesquiterpene
129	(1S,2R,6R,7S,8S)-Copaene	C15H24	204.35		Sesquiterpene
130	(1S,2R,6R,7S,8R)-Copaene	C15H24	204.35		Sesquiterpene
131	(1S,2R,6R,7R,8S)-Copaene	C15H24	204.35		Sesquiterpene
132	(1S,2R,6R,7R,8R)-Copaene	C15H24	204.35		Sesquiterpene
133	(1R,2S,6S,7S,8S)-Copaene	C15H24	204.35		Sesquiterpene
134	(1R,2S,6S,7S,8R)-Copaene	C15H24	204.35		Sesquiterpene
135	(1R,2S,6S,7R,8S)-Copaene	C15H24	204.35		Sesquiterpene
136	(1R,2S,6S,7R,8R)-Copaene	C15H24	204.35		Sesquiterpene
137	(1R,2S,6R,7S,8S)-Copaene	C15H24	204.35		Sesquiterpene
138	(1R,2S,6R,7S,8R)-Copaene	C15H24	204.35		Sesquiterpene
139	(1R,2S,6R,7R,8S)-Copaene	C15H24	204.35		Sesquiterpene
140	(1R,2S,6R,7R,8R)-Copaene	C15H24	204.35		Sesquiterpene
141	(1R,2R,6S,7S,8S)-Copaene	C15H24	204.35		Sesquiterpene
142	(1R,2R,6S,7S,8R)-Copaene	C15H24	204.35		Sesquiterpene
143	(1R,2R,6S,7R,8S)-Copaene	C15H24	204.35		Sesquiterpene
144	(1R,2R,6S,7R,8R)-Copaene	C15H24	204.35		Sesquiterpene
145	(1R,2R,6R,7S,8S)-Copaene	C15H24	204.35		Sesquiterpene

146	(1R,2R,6R,7S,8R)-Copaene	C15H24	204.35		Sesquiterpene
147	(1R,2R,6R,7R,8S)-Copaene	C15H24	204.35		Sesquiterpene
148	(1R,2R,6R,7R,8R)-Copaene	C15H24	204.35		Sesquiterpene

Supplementary Table S2. Structural model quality assessment

Protein model	Ramachandran favored (%)	Ramachandran outliers (%)	Poor rotamers (%)	MolProbity score	Clashscore	GMQE	QMEAN	pLDDT
Ccapobp19a	98.32	0	0	0.5	0	0.79	0.76	NA
Ccapobp19b	97.48	0.77	0	0.88	1.45	0.62	0.64	NA
Ccapobp19d-1	99.29	0	0	0.78	0.94	NA	NA	84.69
Ccapobp28a	100	0	0	0.8	1.04	0.75	0.73	NA
Ccapobp44a	97.56	0	0	0.59	0	NA	NA	73.19
Ccapobp49a	98.41	0.4	0.89	0.67	0.5	0.52	0.48	NA
Ccapobp56d	97.44	0.85	0	0.61	0	NA	NA	82.25
Ccapobp56h	100	0	0	0.85	1.27	NA	NA	84.25
Ccapobp69a	97.37	0	0.96	0.62	0	NA	NA	84.81
Ccapobp83a	100	0	1.79	1.09	1.53	0.89	0.83	NA

Ccapobp84a-2	94.12	0.65	1.46	1.02	0	NA	NA	73.44
Ccapobp99a	100	0	0	0.5	0	0.66	0.65	NA
Ccapobp99c	99.23	0	0.88	0.78	0.94	NA	NA	90
Ccapobp99d	98.45	0	0	0.5	0	NA	NA	77.31
CcOR7a	96.03	0	0.85	0.77	0	NA	NA	89.44
Ccor59b	97.7	0.33	0	0.57	0	NA	NA	81.44
Ccor83b	97.24	0.42	0	0.78	0.4	NA	NA	80.5
Ccor85b	98.3	0	0.8	0.5	0	NA	NA	86.69

Supplementary Table S3. Values of the binding energy (in Kcal/mol) of the sixteen Trimedlure isomers with the fourteen OBPs

	Ccapobp99a	Ccapobp56d	Ccapobp19a	Ccapobp44a	Ccapobp28a	Ccapobp19b	Ccapobp83b	Ccapobp84a	Ccapobp19d-1	Ccapobp49a	Ccapobp56h	Ccapobp69a	Ccapobp99c	Ccapobp99d
1*R*,2*R*,4*R* *Trimedlure B2*	-6.84	-6.74	-6.86	-6.95	-6.06	-5.79	-6.65	-5.67	-6.09	-7.3	-6.23	-7.47	-7.16	-6.77
1*R*,2*R*,4*S* *Trimedlure C*	-7.11	-6.71	-6.87	-7.02	-6.1	-5.51	-6.46	-5.54	-5.91	-6.77	-6.15	-7.22	-7.28	-6.95
1*R*,2*R*,5*R* *Trimedlure B1*	-6.7	-7.06	-7.42	-6.96	-6.47	-5.48	-6.4	-5.85	-5.87	-7.14	-6.13	-7.35	-7.34	-6.66

1*R*,2*R*,5*S* Trimedlure A	-7.11	-6.83	-5.98	-7.12	-6.48	-5.62	-6.42	-6	-5.9	-7.12	-6.03	-7.12	-7.17	-7.12
1*R*,2*S*,4*R* Trimedlure X	-7.16	-6.96	-7.26	-7.18	-6.84	-5.59	-6.38	-5.85	-5.95	-7.18	-5.96	-7.21	-7.19	-7.01
1R,2S,4S Trimedlure W	-7.03	-7.21	-7.11	-6.59	-6.53	-5.56	-6.88	-5.58	-6.02	-6.87	-6.4	-7.21	-7.23	-6.72
1R,2S,5R Trimedlure Y	-7.27	-7.11	-7.5	-6.96	-6.13	-5.66	-6.99	-5.74	-5.9	-7.23	-6.41	-7.33	-7.74	-6.78
1R,2S,5S Trimedlure V	-7.13	-7.04	-6.23	-7.29	-6.06	-5.88	-6.8	-5.6	-5.94	-7.14	-5.96	-7.04	-6.93	-7.02
1S,2R,4R Trimedlure W	-7.4	-7.17	-7.51	-6.85	-6.02	-5.82	-6.99	-5.39	-6.19	-7.38	-6.55	-7	-7.69	-6.87
1S,2R,4S Trimedlure X	-7.11	-7.06	-7.65	-6.75	-6.31	-5.7	-7.02	-5.5	-5.97	-6.97	-6.27	-7.31	-7.66	-7.13
1S,2R,5R Trimedlure V	-7.11	-7.23	-7.09	-6.77	-6.36	-5.71	-6.96	-5.83	-5.93	-7.27	-6.21	-7.24	-7.4	-6.73
1S,2R,5S Trimedlure Y	-7.21	-6.93	-7.43	-7.48	-6.21	-5.81	-6.85	-5.64	-5.87	-7.17	-6.35	-7.31	-7.44	-6.94
1S,2S,4R Trimedlure C	-7.34	-7	-7.17	-6.97	-6.15	-5.9	-7.06	-5.63	-6.25	-7.15	-6.44	-7.35	-7.23	-6.98
1S,2S,4S Trimedlure B2	-4.49	-4.37	-4.24	-4.17	-3.98	-3.4	-4.32	-3.97	-3.8	-4.83	-3.98	-4.13	-4.13	-6.75
1S,2S,5R Trimedlure A	-4.8	-4.68	-4.81	-4.72	-4.47	-3.69	-4.75	-4.53	-3.98	-5.69	-3.84	-4.72	-4.56	-6.91
1S,2S,5S Trimedlure B1	-5.61	-5.54	-5.49	-5.82	-5.23	-4.46	-5.56	-5.3	-5.16	-6.06	-5.15	-5.34	-5.74	-6.72

Supplementary Table S4. Values of the binding energy (in Kcal/mol) of the fourteen OBPs with commercial attractants, fermented-origin compounds, and semiochemicals.

	Ccapo bp99a	Ccapo bp56d	Ccapo bp19a	Ccapo bp44a	Ccapo bp28a	Ccapo bp19b	Ccapo bp83b	Ccapo bp84a	Ccapo bp19d-1	Ccapo bp49a	Ccapo bp56h	Ccapo bp69a	Ccapo bp99c	Ccapo bp99d
Attractants														
1-Octen-3-ol	-4.75	-4.63	-4.55	-4.63	-4.23	-3.55	-4.68	-4.44	-3.79	-5.19	-3.72	-4.5	-4.58	-4.1
2,4-Dimethylpyrimidine	-4.61	-4.42	-4.49	-4.43	-4.43	-3.93	-4.3	-4.32	-3.77	-4.9	-3.73	-4.27	-4.41	-3.89
2,6-Dimethylpyrazine	-4.79	-4.26	-4.4	-4.11	-4.52	-3.93	-4.2	-4.18	-3.68	-4.38	-3.74	-4.21	-4.36	-3.92
Acetamide	-3.21	-2.89	-3.68	-2.89	-3.07	-3.34	-3.21	-3.16	-2.91	-3.48	-3.15	-2.91	-3.47	-3.66
Acetic acid	-3.02	-3.06	-3.81	-3.05	-3.17	-3.65	-3.42	3.22	-3.2	-3.49	-2.87	-2.7	-3.16	-3.3
Ammonium acetate	-3.2	-2.92	-3.6	-2.75	-2.65	-2.84	-3.42	-2.95	-2.71	-3.17	-2.58	-2.47	-3.03	-3.07
Eugenol	-5.73	-5.01	-5.96	-5.49	-5.36	-4.69	-5.32	-5.53	-4.64	-6.38	-4.5	-5.56	-5.26	-5.01
Indole	-5.36	-4.91	-6.32	-5.06	-5.18	-4.12	-5.13	-4.57	-4.94	-5.28	-4.58	-5.57	-4.82	-4.54
Methyl eugenol	-5.51	-5.49	-6.18	-5.69	-5.64	-4.67	-5.78	-5.29	-4.96	-6.39	-4.75	-5.54	-5.56	-5.29
N-vinylimidazole	-3.92	-3.4	-3.4	-3.44	-3.52	-2.78	-3.54	-3.36	-3.12	-4.24	-2.92	-3.27	-3.39	-3.36

p-Cresol	-4.69	-4.54	-4.87	-4.49	-4.76	-3.64	-4.77	-4.48	-4.39	-4.62	-4.17	-4.73	-4.79	-4.19
Phenol	-4.29	-4.33	-4.39	-4.22	-4.5	-4.07	-4.84	-4.01	-4.06	-4.63	-4.16	-4.27	-4.28	-3.82
Propylen glycol	-3.15	-3.1	-3.54	-3.12	-2.75	-2.71	-3	-3.06	-2.91	-3.73	-2.77	-2.56	-2.87	-2.89
Putrescine	-5.31	-4.52	-5.4	-4.81	-5.16	-5.85	-5.41	-5.31	-6.23	-6.74	-4.96	-8.11	-5.39	-5.03
Ceralure B1	-6.63	-6.16	-5.62	-7.12	-5.84	-5.43	-6.68	-5.43	-5.58	-6.74	-5.77	-7.27	-5.63	-6.79
Trans-siglure	-6.73	-6.91	-6.42	-6.58	-5.14	-5.18	-6.44	-5.39	-5.6	-6.48	-5.5	-6.91	-6.73	-6.6
Trimethylamine	-3.2	-2.85	-2.68	-3.15	-3.55	-3.54	-3.41	-2.92	-3.6	-3.4	-3.21	-4.13	-3.17	-6.9
1*R*,2*R*,4*S* *Trimedlure C*	-7.11	-6.71	-6.87	-7.02	-6.1	-5.51	-6.46	-5.54	-5.91	-6.77	-6.15	-7.22	-7.28	-6.95
**Fermented-food compounds**														
1,2-Propanediol diacetate	-4.71	-4.1	-4.31	-4.71	-4.63	-3.88	-4.76	-4.39	-4.25	-5.53	-3.95	-4.16	-4.51	-4.33
1-Dodecanol	-5.64	-4.67	-5.23	-5.01	-4.59	-3.8	-5.41	-4.48	-4.69	-5.73	-4.36	-5.19	-5.06	-4.56
1-(Acetyloxy)-2-butanone	-4.23	-4.01	-4.19	-4.39	-4.17	-3.42	-4.16	-4.02	-3.72	-4.87	-3.47	-3.78	-3.98	-4.24
2-Phenethyl acetate	-6.22	-5.73	-5.86	-5.68	-5.48	-4.33	-5.47	-4.85	-4.74	-6.45	-4.6	-5.5	-5.85	-5.09
3-(Methylthio)propyl acetate	-4.31	-3.75	-4.01	-4.13	-4.02	-3.04	-3.97	-3.48	-3.47	-4.87	-3.17	-3.67	-4.1	-3.71
3-Pentan-2-ol	-4	-3.74	-3.71	-3.83	-3.97	-3.93	3.78	-4.01	-3.59	-4.44	-3.54	-3.67	-3.6	-3.66

4-Decanol	-5.19	-4.89	-5.31	-5.27	-4.28	-3.72	-5.19	-4.23	-4.14	-5.73	-4.21	-4.9	-5.12	-4.82
Acetoin	-3.3	-3.53	-3.71	-3.74	-3.92	-3.23	-4.52	-3.67	-3.18	-3.69	-3.2	-3.12	-3.28	-3.41
Alloaroma dendrene	-6.59	-6.35	-5.62	-7.28	-7.32	-5.96	-7.39	-5.66	-6.47	-7.6	-6.7	-8.4	-7.7	-7.6
Benzyl alcohol	-4.51	-4.53	-4.64	-4.62	-4.29	-3.51	-4.82	-4.48	-4.31	-4.94	-3.83		-4.63	-4.13
Butyl hexanoate	-5.7	-4.68	-5.14	-5.58	-4.47	-3.79	-4.76	-3.91	-3.99	-5.72	-3.91	-4.93	-5.41	-4.88
Caryophyll ene	-6.85	-6.93	-5.27	-7.42	-6.98	-6.08	-7.48	-5.7	-6.51	-7.74	-6.52	-8.25	-6.84	-7.66
Ciclohexan one	-4.3	-4.41	-4.33	-3.95	-4.07	-3.32	-4.59	-4.2	-4.08	-4.32	-3.78	-4.32	-4.04	-3.9
Dodecanal	-6.04	-4.96	-5.57	-4.91	-4.65	-3.89	-5.39	-3.69	-4.78	-6.27	-4.6	-5.07	-5.1	-5.48
Ethyl benzoate	-5.48	-5.14	-5.46	-5.45	-4.9	-4.3	-5.55	-5.1	-4.85	-5.63	-4.37	-5.14	-5.59	-4.91
Ethyl hexanoate	-4.98	-4.27	-4.51	-5.44	-4.43	-3.31	-4.37	-4.44	-3.91	-5.3	-3.39	-4.17	-4.66	-4.26
Hexanoic acid	-4.42	-3.58	-4.24	-5	-4.55	-3.12	-3.43	-4.04	-3.38	-4.85	-3.65	-2.89	-4.23	-4.4
Isobutyl acetate	-4.44	-4.1	-4.19	-4.2	-4.36	-3.45	-4.23	-4.04	-3.8	-4.85	-3.51	-3.86	-3.91	-3.81
Isopentyl acetate	-4.63	-4.33	-4.31	-4.63	-4.32	-3.56	-4.43	-4.35	-3.81	-5.2		-4.17	-4.52	-4.12
Methionol	-3.06	-3.01	-3	-3.18	-3.11	-2.43	-3.23	-3.63	-2.85	-3.59	-3.05	-3.09	-3.23	-3.06
Methyl pentanoate	-4.06	-3.63	-4.35	-4.29	-3.79	-3.3	-3.78	-3.83	-3.74	-4.68	-3.31	-3.58	-3.86	-3.55
Phenylethy l alcohol	-4.92	-4.62	-5.8	-4.93	-4.57	-3.73	-4.98	-4.77	-4.48	-5.29	-4.09	-4.74	-5.04	-4.41
Santolina triene	-5.64	-5.54	-5.83	-4.66	-4.62	-3.88	-5.3	-4.53	-4.58	-5.44	-4.5	-5.59	-5.11	-4.99
ß-pinene	-6.32	-5.93	-5.52	-5.56	-5.08	-4.43	-6.42	-4.73	-5.37	-5.81	-5.24	-6.46	-5.54	-5.66
Styrene	-5	-4.84	-5.03	-4.44	-4.6	-3.56	-4.65	-4.83	-4.55	-5.09	-4.06	-4.83	-4.7	-4.78

trans-ß-Damascone	-6.79	-7.13	-6.13	-7.07	-7.01	-5.73	-6.66	-5.72	-5.95	-6.99	-6.34	-7.53	-7.56	-7.01
Semiochemicals														
(*E*)-2-Hexenal	-4.4	-3.78	-4.1	-3.93	-3.63	-3.01	-3.97	-4.31	-3.39	-4.74	-3.5	-3.64	-3.94	-3.81
3-Methylbutanal	-3.86	-3.49	-3.28	-3.41	-3.5	-2.93	-4.39	-3.59	-3.26	-3.95	-3.58	-3.35	-3.44	-3.17
3-Methylbutanol	-3.32	-3.53	-3.94	-3.54	-3.52	-2.77	-3.67	-3.78	-3.24	-4.05	-3.58	-3.49	-3.52	-3.3
α-Copaene	-8.33	-8.53	-6.15	-7.41	-7.07	-6.26	-7.61	-5.84	-6.81	-7.97	-7.31	-8.46	-6.8	-7.92
Decanal	-5.32	-5.11	-4.94	-4.53	-4.26	-3.53	-4.87	-3.39	-4.04	-5.93	-3.96	-4.61	-4.63	-4.79
Ethyl acetate	-3.39	-3.41	-3.86	-3.37	-3.6	-3.34	-3.19	-3.51	-3.18	-3.86	-3.1	-3.13	-3.15	-3.12
Geranyl acetate	-6.45	-6.58	-6.85	-5.87	-5.87	-4.67	-6.17	-5.34	-5.3	-7.03	-5.3	-6.1	-6.16	-6.01
Hexanal	-3.93	-3.41	-3.55	-3.64	-3.4	-2.92	-3.72	-3.93	-3.1	-4.49	-3.43	-3.52	-3.72	-3.58
Hexanol	-3.71	-3.58	-3.56	-3.78	-3.7	-2.91	-3.7	-4.19	-3.31	-4.4	-3.28	-3.6	-3.8	-3.6
Isomenthone	-6.55	-6.23	-6.27	-6	-5.76	-4.97	-6.66	-5.73	-5.6	-6.01	-5.29	-6.3	-6.15	-5.68
Linalool	-5.86	-5.84	-5.93	-5.54	-4.59	-4.15	-5.52	-4.56	-4.67	-6.3	-4.97	-5.74	-5.86	-5.04
Methyl hexanoate	-4.34	-4.03	-4.43	-4.64	-4.03	-3.23	-4.09	-4.25	-3.85	-5.03	-3.46	-3.95	-4.27	-3.93
(*E*)-2-Methyl-3-penten-2-ol	-7.05	-7.04	-4.5	-6.72	-6.03	-5.64	-6.63	-5.29	-6.01	-6.86	-6.3	-6.3	-6.96	-4.08
(*E*,*Z*)-3,6-Octadien-1-ol	-7.22	-7.46	-7	-6.69	-6.47	-5.58	-6.72	-5.84	-6	-7	-6.15	-7.33	-7.26	-4.44
Methyl (*E*)-N-	-6.94	-6.1	-5.62	-6.79	-6.21	-5.72	-6.36	-5.69	-5.88	-6.93	-5.94	-7.04	-6.52	-4.92

hydroxybe nzimidate														
E-*E*-α-farnesene	-7.83	-7.25	-7.96	-6.18	-6.55	-5.51	-6.94	-5.41	-6.33	-7.59	-6.31	-7.11	-6.84	-6.84
E-β-farnesene	-7.59	-7.09	-7.75	-6.47	-5.89	-5.12	-6.68	-4.89	-5.91	-7.09	-5.89	-6.57	-6.42	-6.78

Supplementary Table S5. Values of the binding energy (in Kcal/mol) of the fourteen OBPs with stereoisomers of α-copaene

	Ccapo bp99a	Ccapo bp56d	Ccapo bp19a	Ccapo bp44a	Ccapo bp28a	Ccapo bp19b	Ccapo bp83b	Ccapo bp84a	Ccapo bp19d -1	Ccap obp4 9a	Ccap obp5 6h	Ccap obp6 9a	Ccap obp9 9c	Ccap obp9 9d
(1S,2S,6S,7S,8S)-Copaene	-7.63	-8.31	-7.2	-7.16	-6.99	-6.21	-8.03	-6.22	-7.02	-7.7	7.16	-8.64	-7.53	-7.86
(1S,2S,6S,7S,8R)-Copaene	-8.22	-8.53	-6.15	-7.41	-7.07	-6.26	-7.61	-5.84	-6.82	-7.97	-7.31	-8.46	-6.8	-7.92
(1S,2S,6S,7R,8S)-Copaene	-8.25	-8.59	-7.79	-7.24	-7.2	-6.33	-7.59	-6.15	-7.2	-7.66	-7.4	-8.78	-6.81	-7.84
(1S,2S,6S,7R,8R)-Copaene	-7.67	-8.12	-6.29	-7.26	-6.98	-5.92	-7.49	-5.76	-7.01	-7.96	-6.85	-8.42	-6.9	-8.01
(1S,2S,6R,7S,8S)-Copaene	-8.26	-8.59	-7.79	-7.24	-7.21	-6.33	-7.59	-6.14	-7.21	-7.66	-7.4	-8.78	-6.84	-7.84
(1S,2S,6R,7S,8R)-Copaene	-7.67	-8.19	-6.29	-7.26	-6.98	-5.91	-7.49	-5.76	-7.01	-7.96	-6.84	-8.41	-6.9	-8.01
(1S,2S,6R,7R,8S)-Copaene	-8.26	-8.6	-7.79	-7.24	-6.96	-6.33	-8.01	-5.96	-7.21	-7.66	-7.4	-8.78	-6.81	-8.01
(1S,2S,6R,7R,8 R)-Copaene	-7.67	-7.83	-6.29	-7.25	-6.97	-5.91	-7.49	-5.76	-7.01	-7.96	-6.84	-8.42	-6.9	-8.01
(1S,2R,6S,7S,8S)-Copaene	-7.63	-8.3	-7.2	-7.16	-6.99	-6.21	-8.03	-6.22	-7.02	-7.7	7.16	-8.64	-7.53	-7.86
(1S,2R,6S,7S,8R)-Copaene	-8.21	-8.53	-6.16	-7.41	-7.07	-6.26	-7.61	-5.84	-6.81	-7.97	-7.31	-8.4	-6.8	-7.92
(1S,2R,6S,7R,8S)-Copaene	-8.25	-8.59	-7.79	-7.24	-6.95	-6.33	-8.01	-6.13	-7.21	-7.66	-7.4	-8.78	-6.82	-7.84
(1S,2R,6S,7R,8 R)-Copaene	-8.01	-8.39	-6.03	-7.22	-6.97	-6.12	-7.72	-5.82	-7.16	-8.05	-6.94	-8.47	-6.89	-7.97
(1S,2R,6R,7S,8S)-Copaene	-7.87	-7.85	-6.33	-7.23	-7.08	-6.03	-7.68	-5.8	-7.2	-7.59	-7.15	-8.48	-7.41	-8.04

(1S,2R,6R,7S,8R)-Copaene	-8.23	-7.86	-6.58	-7.43	-7.11	-6.34	-7.58	-5.81	-7.03	-7.61	-7.03	-8.73	-7.33	-8.11
(1S,2R,6R,7R,8S)-Copaene	-8.25	-8.6	-7.79	-7.24	-6.95	-6.33	-8.01	-6.15	-7.21	-7.66	-7.41	-8.78	-6.81	-7.84
(1S,2R,6R,7R,8R)-Copaene	-7.67	-7.83	-6.28	-7.25	-6.97	-5.91	-7.49	-5.76	-7.01	-7.96	-6.85	-8.41	-6.9	-8.01
(1R,2S,6S,7S,8S)-Copaene	-7.64	-8.32	-7.2	-7.16	-6.99	-6.21	-8.02	-6.22	-7.02	-7.7	-7.16	-8.57	-7.55	-7.86
(1R,2S,6S,7S,8R)-Copaene	-8.22	-8.53	-6.16	-7.41	-7.07	-6.26	-7.61	-5.84	-6.81	-7.97	-7.31	-8.48	-6.8	-7.92
(1R,2S,6S,7R,8S)-Copaene	-7.87	-7.83	-6.33	-7.23	-7.08	-6.03	-7.68	-5.77	-7.2	-7.59	-7.15	-8.44	-7.42	-8.04
(1R,2S,6S,7R,8R)-Copaene	-8.13	-8.21	-6.85	-7.44	-7.3	-6.48	-7.78	-5.89	-7.16	-7.8	-7	-8.44	-6.83	-7.74
(1R,2S,6R,7S,8S)-Copaene	-8.26	-8.59	-7.79	-7.24	-7.11	-6.33	-8.01	6.15	-7.21	-7.66	-7.4	-8.78	-6.81	-7.84
(1R,2S,6R,7S,8R)-Copaene	-7.67	-7.83	-6.29	-7.25	-6.97	-5.92	-7.49	-5.76	-7.01	-7.96	-6.86	-8.42	-6.9	-8.01
(1R,2S,6R,7R,8S)-Copaene	-8.26	-8.6	-7.79	-7.24	-7.21	-6.32	-8.01	-6.15	-7.21	-7.66	-7.41	8.78	-6.83	-7.84
(1R,2S,6R,7R,8R)-Copaene	-7.67	-7.83	-6.29	-7.26	-6.97	-5.92	-7.49	-5.76	-7.01	-7.96	-6.86	-8.42	-6.9	-8.01
(1R,2R,6S,7S,8S)-Copaene	-8.16	-8.11	-6.6	-7.28	-7.23	-6.55	-7.72	-5.87	-7.34	-7.82	-7.15	-8.44	-6.88	-7.92
(1R,2R,6S,7S,8R)-Copaene	-8.31	-8.48	-6.32	-7.46	-7.36	-6.2	-7.93	-5.9	-6.73	-7.79	-7.14	-8.55	-6.91	-7.75
(1R,2R,6S,7R,8S)-Copaene	-7.87	-7.84	-6.33	-7.23	-7.08	-6.03	-7.69	-5.77	-7.2	-7.59	-7.15	-8.48	-7.42	-8.02
(1R,2R,6S,7R,8R)-Copaene	-8.11	-8.15	-6.77	-7.38	-7.36	-6.46	-7.85	-5.9	-7.15	-7.79	-6.97	-8.42	-6.82	-7.66
(1R,2R,6R,7S,8S)-Copaene	-8.26	-8.59	-7.79	-7.24	-6.95	-6.33	-8.01	-6.14	-7.2	-7.66	-7.4	-8.78	-6.81	-7.84
(1R,2R,6R,7S,8R)-Copaene	-7.66	-7.83	-6.29	-7.26	-6.98	-5.92	-7.49	-5.76	-7.01	-7.96	-6.85	-8.42	-6.9	-8.01

(1R,2R,6R,7R,8S)-Copaene	-8.15	-8.15	-6.56	-7.31	-7.22	-6.56	-7.72	-6.4	-7.32	-7.8	-7.17	-8.46	-6.84	-7.94
(1R,2R,6R,7R,8R)-Copaene	-8.13	-8.21	-6.85	-7.43	-7.29	-6.49	-7.78	-5.89	-7.16	-7.8	-7	-8.42	-6.84	-7.74

Supplementary Table S6. Values of the binding energy (in Kcal/mol) of the fourteen OBPs with stereoisomers of some sesquiterpenes

IUPAC name	Common name	Ccapobp99a	Ccapobp56d	Ccapobp19a	Ccapobp44a	Ccapobp28a	Ccapobp19b	Ccapobp83a	Ccapobp84a-2	Ccapobp19d-1	Ccapobp49a	Ccapobp56h	Ccapobp69a	Ccapobp99c	Ccapobp99d
E-beta-farnesene	E-beta-farnesene	-7.59	-7.09	-7.75	-6.47	-5.89	-5.12	-6.68	-4.89	-5.91	-7.09	-5.89	-6.57	-6.42	-6.78
Z-beta-farnesene	Z-beta-farnesene	-7.64	-7.03	-7.56	-6.28	-5.99	-4.97	-6.51	-4.85	-5.99	-6.87	-5.95	-6.85	6.11	-6.74
E-E-α-farnesene	E-E-α-farnesene	-7.83	-7.25	-7.96	-6.18	-6.55	-5.51	-6.94	-5.41	-6.33	-7.59	-6.31	-7.11	-6.84	-6.84
E-Z-α-farnesene	E-Z-α-farnesene	-7.69	-7.13	-7.8	-6.2	-6.13	-5.15	-7.09	-5.01	-6.18	-7.95	-6.48	-7.14	-6.68	-7.17
Z-E-α-farnesene	Z-E-α-farnesene	-8.16	-7.37	-7.91	-6.85	-6.07	-5.26	-7.04	-5.45	-6.44	-7.53	-6.09	-6.76	-7.15	-6.77
Z,Z-α-farnesene	Z,Z-α-farnesene	-7.97	-7.19	-7.74	-6.34	-6.06	-5.24	-7.02	-5.5	-6.28	-7.39	-6.28	-7.2	-6.55	-6.84
(1*E*,5*E*,8*S*)-1,5-dimethyl-8-(prop-1-en-2-yl)cyclodeca-1,5-diene	(S)-Germacrene A	-7.13	-7.35	-5.58	-7.41	-7.21	-6.15	-7.27	-5.5	-6.27	-7.67	-7.18	-8.31	-6.81	-7.59
(1*E*,5*E*,8R)-1,5-dimethyl-8-(prop-1-en-2-	(R)-Germacrene A	-6.69	-7	-5	-6.76	-6.88	-5.73	-7.15	-5.63	-6.45	-7.21	-6.83	-8.25	-6.81	-7.71

yl)cyclodeca-1,5-diene															
(1*E*,5*E*)-1,5-dimethyl-8-propan-2-ylidenecyclodeca-1,5-diene	Germacrene B	-6.5	-6.43	-5.24	-6.87	-6.76	-6.06	-7.31	-5.69	-6.49	-7.3	-7.18	-7.86	-6.89	-7.68
(1*E*,6*E*,8*S*)-1-methyl-5-methylidene-8-propan-2-ylcyclodeca-1,6-diene	Germacrene C	-7.25	-8.05	-7.02	-7.17	-6.95	-5.94	-7.33	-5.75	-6.96	-7.43	-7.04	-8.28	-7.02	-7.78
(1*E*,5*E*,8*S*)-1,5-dimethyl-8-(prop-1-en-2-yl)cyclodeca-1,5-diene	(S)-Germacrene D	6.66	-6.58	-5.65	-7.38	-6.76	-5.65	-7.35	-5.58	-5.99	-7.4	-6.73	-8.38	-7.63	-7.62
(1*E*,5*E*,8*R*)-1,5-dimethyl-8-(prop-1-en-2-yl)cyclodeca-1,5-diene	(R)-Germacrene D	-7.4	-6.68	-6.43	-7.59	-7.11	-6.06	-7.54	-5.66	-6.28	-7.59	-6.92	-8.28	-7.62	-7.76
(1E,6E)-1,5-dimethyl-8-(prop-1-en-2-yl)cyclodeca-1,6-diene	Germacrene E	-6.97	-6.56	-5.48	-7.67	-7.02	-5.97	-7.5	-5.79	-6.51	-7.54	-6.56	-8.22	-7.63	-7.49
(+)-α: (*S*)-1-Isopropyl-6-methyl-3-(propan-2-	(+)-α-Elemene	-7.55	-6.98	-8	-7.13	-6.66	-6.07	-7.3	-5.62	-6.44	-7.4	-6.6	-8.35	-7.36	-8.26

ylidene)-6-vinylcyclohex-1-ene															
(−)-β: (1*S*,2*S*,4*R*)-1-Methyl-2,4-di(prop-1-en-2-yl)-1-vinylcyclohexane	(−)-β-Elemene	-7.49	-7.61	-7.32	-7.11	-6.69	-5.83	-7.09	-5.55	-6.31	-7.1	-6.72	-7.91	-7.43	-7.74
(−)-γ: (3*R*,4*R*)-1-Isopropyl-4-methyl-3-(prop-1-en-2-yl)-4-vinylcyclohex-1-ene	(−)-γ-Elemene	-7.77	-6.51	-6.2	-7.12	-6.64	-5.65	-7.01	-5.39	-6.21	-7.27	-6.49	-7.99	-6.74	-7.31
(−)-δ: (3*R*,4*R*)-1-Isopropyl-4-methyl-3-(prop-1-en-2-yl)-4-vinylcyclohex-1-ene	(−)-δ-Elemene	-7.82	-6.52	-6.17	-7.12	-6.64	-5.65	-7.01	-5.38	-6.21	-7.27	-6.47	-7.99	-6.68	-7.23
(1S,5S,6S)-2,6-dimethyl-6-(4-methylpent-3-en-1-yl)bicyclo[3.1.1]hept-2-ene	α-cis-Bergamotene	-8.04	-7.77	-7.47	-7.4	-6.6	-5.79	-7.29	-5.6	-6.43	-8.37	-6.74	-8.21	-7.06	-7.51

(1S,5S,6S)-6-methyl-2-methylene-6-(4-methylpent-3-en-1-yl)bicyclo[3.1.1]heptane	β-cis.Bergamotene	-7.68	-7.75	-7.5	-7.38	-6.76	-5.73	-7.35	-5.63	-6.51	-8.41	-6.7	-8.28	-6.95	-7.55
(1S,5S,6R)-2,6-dimethyl-6-(4-methylpent-3-en-1-yl)bicyclo[3.1.1]hept-2-ene	α-trans-Bergamotene	-7.93	-7.9	-7.33	-7.41	-7.03	-5.82	-7.78	-5.71	-6.66	-8.23	-6.66	-8.09	-7.44	-7.53
(1S,5S,6R)-6-methyl-2-methylene-6-(4-methylpent-3-en-1-yl)bicyclo[3.1.1]heptane	β-trans-Bergamotene	-8.14	-7.64	-7.27	-7.53	-7.05	-5.69	-7.88	-5.8	-6.59	-8.09	-6.63	-8	-7.37	-7.62
2,6,6,9-Tetramethyl-1,4-8-cycloundecatriene	Humulene	-6.76	-6.47	-5.28	-6.75	-6.89	-5.95	-7.34	-5.82	-6.41	-7.5	-6.54	-8.06	-7.61	-7.94
(1S,3aS,3bR,6aS,6bR)-1-Isopropyl-3a-methyl-6-methylenedecahydrocyclobu	β-bourbonene	-7.31	-6.56	-5.81	-7.71	-7.26	-5.96	-7.36	-6.04	-6.26	-7.58	-6.72	-8.58	-7.35	-7.87

ta[1,2:3,4]di[5]annulene															
(1aR,4R,4aR,7bS)-1,1,4,7-Tetramethyl-1a,2,3,4,4a,5,6,7b-octahydrocyclopropa[e]azulene	α-Gurjunene	-7.18	-6.7	-6.18	-7.18	-7.08	-6.09	-7.39	-5.56	-6.19	-7.38	-6.58	-8.32	-7.01	-7.75
(1aR,4R,4aR,7bR)-1,1,4-trimethyl-7-methylenedecahydro-1H-cyclopropa[e]azulene	β-Gurjunene	-7.04	-6.7	-5.93	-7.37	-7.12	-5.97	-7.51	-5.5	-6.25	-7.4	-6.6	-8.38	-7.33	-7.69
(1R,3aR,4R,7S)-7-isopropyl-1,4-dimethyl-1,2,3,3a,4,5,6,7-octahydroazulene	γ-Gurjunene	-8.5	-7.67	-7.28	-7.1	-6.99	-6.1	-7.44	-6.07	-6.72	-7.89	-7.55	-8.56	-7.32	-8.14
(1*S*,4a*R*,8a*R*)-4,7-dimethyl-1-(propan-2-yl)-1,2,4a,5,6,8a-hexahydronaphthalene	α-Cadinene	-7.33	-7.33	-7.22	-7.39	-7.21	-6.1	-7.77	-5.95	-6.63	-7.64	-7.06	-8.59	-7.59	-7.58
(1S,4aR,8aS)-1-isopropyl-	β-Cadinene	-7.42	-7	-6.34	-7.42	-7.3	-6.01	-7.24	-5.93	-6.36	-7.73	-7.12	-8.1	-7.66	-8.21

4,7-dimethyl-1,2,4a,5,8,8a-hexahydronaphthalene															
(1S,4aR,8aR)-1-isopropyl-7-methyl-4-methylene-1,2,3,4,4a,5,6,8a-octahydronaphthalene	γ-Cadinene	-7.44	-6.95	-6.94	-7.5	-7.45	-5.65	-7.43	-6.07	-6.47	-7.67	-7.15	-7.99	-7.75	-8
(1S,8aR)-1-isopropyl-4,7-dimethyl-1,2,3,5,6,8a-hexahydronaphthalene	δ-Cadinene	-7.07	-7.81	-6.91	-7.17	-7.19	-6.55	-7.4	-6.09	-6.3	-7.65	-6.93	-8.64	-7.86	-7.66
(1R,4aR,8aS)-1-isopropyl-4,7-dimethyl-1,2,4a,5,6,8a-hexahydronaphthalene	α-Muurolene	-7.32	-6.92	-6.91	-7.24	-7.32	-6.21	-7.28	-5.9	-6.37	-7.38	-6.92	-8.47	-7.58	-7.82
(1R,4aR,8aS)-1-isopropyl-7-methyl-4-methylene-1,2,3,4,4a,5,6,8a-octahydronaphthalene	γ-Muurolene	-7.98	-7.05	-6.96	-7.28	-7.49	-6.36	-7.42	-5.96	-6.26	-7.77	-6.87	-8.45	-7.52	-7.76
(1*R*,5*S*,6*R*,7*S*,10*R*)-4,10-	α-Cubebene	-7.57	-6.78	-6.64	-7.24	-6.85	-5.71	-7.29	-5.77	-6.19	-7.33	-6.53	-8.19	-7.46	-7.9

Dimethyl-7-propan-2-yltricyclo[4.4.0.0^{1,5}]dec-3-ene															
(3aS,3bR,4S,7R,7aR)-4-isopropyl-7-methyl-3-methyleneoctahydro-1H-cyclopenta[1,3]cyclopropa[1,2]benzene	β-Cubebene	-7.74	-7.65	-7.57	-7.12	-6.8	-5.88	-7.38	-5.97	-6.64	-7.63	-7.33	-8.41	-7.63	-8.07
(1R,3aS,7S,8aR)-1,4,9,9-Tetramethyl-2,3,6,7,8,8a-hexahydro-1H-3a,7-methanoazulene	α-Patchoulene	-6.99	-6.64	-4.83	-7.37	-6.82	-5.89	-7.61	-5.38	-6.43	-7.81	-6.38	-8.54	-5.91	-7.57
(1R,5R,8S)-1,5,11,11-tetramethyltricyclo[6.2.1.02,6]undec-2(6)-ene	β-Patchoulene	-6.92	-7.81	-5.76	-7.46	-6.99	-6.12	-7.68	-5.79	-6.63	-7.81	-6.63	-8.37	-6.45	-7.38
(1*S*,4*S*,7*R*)-1,4-Dimethyl-7-(prop-1-en-2-yl)-1,2,3,4,5,6,7,8-	α-Guaiene	-7.42	-7.26	-5.93	-7.24	-7.27	-6.17	-7.51	-5.97	-6.28	-7.75	-7.26	-8.64	-7.83	-7.92

octahydroazulene															
(1*S*,4*S*)-1,4-Dimethyl-7-(propan-2-ylidene)-1,2,3,4,5,6,7,8-octahydroazulene	β-Guaiene	-6.48	-6.82	-6.36	-7.03	-7.42	-6.08	-7.22	-5.89	-6.29	-7.48	-6.99	-8.07	-7.85	-7.31
(3*S*,3a*S*,5*R*)-3,8-Dimethyl-5-(prop-1-en-2-yl)-1,2,3,3a,4,5,6,7-octahydroazulene	δ-Guaine	-7.54	-7.9	-7.57	-7.37	-7.33	-6.45	-7.52	-6.03	-6.77	-7.57	-7.14	-8.28	-7.74	-7.74
(1R,4S)-1,3,3-trimethylbicyclo[2.2.1]heptan-2-one	1R,4S-Fenchone	-6.52	-6.35	-5.66	-6.08	-5.63	-5.05	-6.7	-4.96	-5.31	-6.48	-5.5	-6.74	-5.86	-5.98
(1S,4R)-1,3,3-trimethylbicyclo[2.2.1]heptan-2-one	1S,4R-Fenchone	-6.29	-6.39	-5.61	-6.03	-5.87	-5.07	-6.32	-4.98	-5.33	-6.28	-5.47	-6.73	-6.17	-5.82
α-Copaene	α-Copaene	-8.33	-8.53	-6.15	-7.41	-7.07	-6.26	-7.61	-5.84	-6.81	-7.97	-7.31	-8.46	-6.8	-7.92
1*R*,2*R*,4*S* *Trimedlure C*	Trimedlure *C*	-7.11	-6.71	-6.87	-7.02	-6.1	-5.51	-6.46	-5.54	-5.91	-6.77	-6.15	-7.22	-7.28	-6.95

Supplementary Table S7. Values of the binding energy (in Kcal/mol) of the sixteen Trimedlure isomers with the four ORs of *C. capitata*.

Trimedlure isomer	Ccor7a	Ccor59b	Ccor83b	Ccor85b
1R,2R,4R Trimedlure B2	-5.75	-4.71	-5.69	-5.38
1R,2R,4S Trimedlure C	-5.71	-4.82	-5.87	-5.73
1R,2R,5R Trimedlure B1	-5.86	-4.51	-5.72	-5.72
1*R*,2*R*,5*S* Trimedlure A	-5.82	-5.09	-5.91	-5.61
1*R*,2*S*,4*R* Trimedlure X	-5.65	-4.77	-5.71	-5.65
1R,2S,4S Trimedlure W	-5.46	-4.85	-5.88	-5.83
1R,2S,5R Trimedlure Y	-5.84	-4.89	-5.7	-5.63
1R,2S,5S Trimedlure V	-5.69	-4.97	-5.75	-5.66
1S,2R,4R Trimedlure W	-5.62	-5.8	-5.85	-5.68
1S,2R,4S Trimedlure X	-5.69	-4.96	-6.6	-7.37
1S,2R,5R Trimedlure V	-5.76	-4.78	-5.87	-5.85
1S,2R,5S Trimedlure Y	-5.7	-5.79	-6	-5.89
1S,2S,4R Trimedlure C	-5.77	-5.42	-5.88	-5.83
1S,2S,4S Trimedlure B2	-5.88	-5.65	-6.8	-5.44
1S,2S,5R Trimedlure A	-5.81	-5.16	-5.79	-6.06
1S,2S,5S Trimedlure B1	-5.86	-6.61	-5.77	-6.12

Supplementary Table S8. Values of the binding energy (in Kcal/mol) of the four ORs with stereoisomers of α-copaene

α-Copaene isomer	Ccor7a	Ccor59b	Ccor83b	Ccor85b
(1S,2S,6S,7S,8S)-Copaene	-6.44	-5.45	-6.7	-6.57
(1S,2S,6S,7S,8R)-Copaene	-6.38	-6.04	-6.73	-6.51
(1S,2S,6S,7R,8S)-Copaene	-6.47	-7.46	-6.84	-6.62
(1S,2S,6S,7R,8R)-Copaene	-5.92	-5.57	-6.69	-6.4
(1S,2S,6R,7S,8S)-Copaene	-6.47	-5.77	-6.84	-6.62
(1S,2S,6R,7S,8R)-Copaene	-5.92	-5.75	-6.69	-6.05
(1S,2S,6R,7R,8S)-Copaene	-6.47	-5.63	-6.84	-6.62
(1S,2S,6R,7R,8R)-Copaene	-5.92	-5.6	-6.69	-6.05
(1S,2R,6S,7S,8S)-Copaene	-6.43	-5.62	-6.7	-6.57
(1S,2R,6S,7S,8R)-Copaene	-6.38	-6.04	-6.73	-6.51
(1S,2R,6S,7R,8S)-Copaene	-6.47	-5.63	-6.84	-6.62
(1S,2R,6S,7R,8R)-Copaene	-6.24	-5.75	-6.67	-6.29
(1S,2R,6R,7S,8S)-Copaene	-6.39	-5.49	-6.89	-6.11
(1S,2R,6R,7S,8R)-Copaene	-6.4	-5.69	-6.89	-6.25
(1S,2R,6R,7R,8S)-Copaene	-6.47	-5.63	-6.84	-6.62
(1S,2R,6R,7R,8R)-Copaene	-6.13	-5.75	-6.69	-6.05
(1R,2S,6S,7S,8S)-Copaene	-6.43	-5.57	-6.7	-6.57
(1R,2S,6S,7S,8R)-Copaene	-6.38	-6.03	-6.73	-6.51
(1R,2S,6S,7R,8S)-Copaene	-6.39	-5.49	-6.89	-6.11
(1R,2S,6S,7R,8R)-Copaene	-6.44	-6.06	-6.96	-6.51
(1R,2S,6R,7S,8S)-Copaene	-6.47	-5.77	-6.84	-6.62
(1R,2S,6R,7S,8R)-Copaene	-6.13	-5.75	-6.69	-6.05
(1R,2S,6R,7R,8S)-Copaene	-6.47	-5.63	-6.84	-6.62
(1R,2S,6R,7R,8R)-Copaene	-6.1	-5.68	-6.69	-6.05

(1R,2R,6S,7S,8S)-Copaene	-6.79	-5.58	-7.01	-6.29
(1R,2R,6S,7S,8R)-Copaene	-6.12	-5.71	-6.85	-6.37
(1R,2R,6S,7R,8S)-Copaene	-6.39	-5.49	-6.89	-6.11
(1R,2R,6S,7R,8R)-Copaene	-6.42	-6.07	-6.96	-6.48
(1R,2R,6R,7S,8S)-Copaene	-6.47	-7.41	-6.84	-6.62
(1R,2R,6R,7S,8R)-Copaene	-6.1	-5.54	-6.69	-6.05
(1R,2R,6R,7R,8S)-Copaene	-6.86	-5.58	-7.03	-6.33
(1R,2R,6R,7R,8R)-Copaene	-6.44	-6.06	-6.97	-6.51

Supplementary Table S9. Values of the binding energy (in Kcal/mol) of the four ORs with stereoisomers of some sesquiterpenes

**Sesquiterpenes**	**Ccor7a**	**Ccor59b**	**Ccor83b**	**Ccor85b**
1S,2S,4R Trimedlure C	-5.77	-5.42	-5.88	-5.83
Caryophyllene	-5.93	-5.46	-6.64	-5.8
α-Copaene	-6.38	-6.04	-6.73	-6.51
Alloaromadendrene	-6.52	-5.55	-6.14	-6.09
E-β-farnesene	-5.55	-5.48	-4.9	-5.44
E-E-α-farnesene	-5.88	-5.31	-5.43	-5.76
E-Z-α-farnesene	-5.66	-5.09	-5.58	-5.57
Z-β-farnesene	-5.85	-4.82	-5.39	-5.33
Z-E-α-farnesene	-6.04	-4.74	-5.42	-5.46
Z,Z-α-farnesene	-5.81	-5.06	-5.76	-5.66
(S)-Germacrene A	-6.44	-5.34	-6.21	-6.15
(R)-Germacrene A	-5.58	-5.58	-5.59	-5.9
Germacrene B	-5.91	-5.82	-5.85	-6.28
Germacrene C	-6.43	-5.32	-5.96	-6.49
(S)-Germacrene D	-5.81	-5.23	-6	-5.99
(R)-Germacrene D	-6.44	-5.35	-6.14	-5.99
Germacrene E	-6.12	-5.3	-5.9	-6.03
(+)-α-Elemene	-6.37	-5.62	-6.67	-5.97
(−)-β-Elemene	-6	-5.13	-5.89	-5.79
(−)-γ-Elemene	-6.13	-5.02	-6.69	-5.88
(−)-δ-Elemene	-6.11	-5.02	-6.7	-5.88
α-cis-Bergamotene	-6.21	-5.95	-6.49	-6.39

β-cis.Bergamotene	-6.13	-5.99	-6.4	-6.36
α-trans-Bergamotene	-6.45	-5.74	-6.2	-6.36
β-trans-Bergamotene	-6.55	-5.86	-6.39	-6.27
Humulene	-5.78	-5.83	-5.75	-6.15
β-bourbonene	-6.51	-5.59	-6.35	-5.84
α-Gurjunene	-5.89	-5.48	-6	-5.91
β-Gurjunene	-5.83	-5.49	-6	-6
γ-Gurjunene	-6.02	-5.77	-6.39	-6.82
α-Cadinene	-6.19	-6.24	-6.27	-6.5
β-Cadinene	-6.23	-7.44	-6.53	-6.5
γ-Cadinene	-6.4	-6.19	-7.78	-6.49
δ-Cadinene	-6.33	-5.57	-6.13	-6.26
α-Muurolene	-6.1	-6.11	-6.28	-6.23
γ-Muurolene	-6.26	-5.98	-6.41	-6.3
α-Cubebene	-6.04	-6.67	-6.99	-6.1
β-Cubebene	-6.27	-5.93	-6.49	-6.5
α-Patchoulene	-6.52	-5.4	-5.9	-5.95
β-Patchoulene	-6.33	-5.6	-6.27	-6.55
α-Guaiene	-5.87	-5.52	-5.61	-6.15
β-Guaiene	-5.85	-5.44	-5.83	-6.22
δ-Guaine	-6.55	-5.65	-6.13	-6.37
1R,4S-Fenchone	-5.51	-4.87	-5.45	-6.48
1S,4R-Fenchone	-5.46	-6.11	-5.71	-6.56

Supplementary Table S10. Values the antennal response (in mV) of the medfly

REP 1		REP 2		REP 3		REP 4		REP 5	
HEXANE	0.05	HEXANE	0.1	HEXANE	0.01	HEXANE	0.1	HEXANE	0.05
LONGIPINENE 0.001	1.811	AROMANDRENE 0.001	1.349	TML 0.001	1.302	β-CARYOPHYLLENE 0.001	1.136	CEDRENE 0.001	0.982
LONGIPINENE 0.01	1.739	AROMANDRENE 0.01	1.205	TML 0.01	1.568	β-CARYOPHYLLENE 0.01	1.219	CEDRENE 0.01	1.039
LONGIPINENE 0.1	1.085	AROMANDRENE 0.1	1.265	TML 0.1	1.785	β-CARYOPHYLLENE 0.1	1.506	CEDRENE 0.1	1.178
LONGIPINENE 1	0.775	AROMANDRENE 1	1.371	TML 1	1.983	β-CARYOPHYLLENE 1	1.43	CEDRENE 1	1.139
HEXANE	0.1	HEXANE	0.05	HEXANE	0.2	HEXANE	0.054	HEXANE	0.1
LONGIFOLENE 0.001	1.305	LONGIFOLENE 0.001	1.235	FARNESENE 0.001	1.502	FARNESENE 0.001	1.129	TML 0.001	1.275
LONGIFOLENE 0.01	1.196	LONGIFOLENE 0.01	1.132	FARNESENE 0.01	1.406	FARNESENE 0.01	0.95	TML 0.01	1.398
LONGIFOLENE 0.1	1.256	LONGIFOLENE 0.1	1.21	FARNESENE 0.1	1.478	FARNESENE 0.1	1.45	TML 0.1	1.562
LONGIFOLENE 1	1.305	LONGIFOLENE 1	1.301	FARNESENE 1	1.45	FARNESENE 1	1.655	TML 1	1.897
HEXANE	0.1	HEXANE	0.105	HEXANE	0	HEXANE	0.23	HEXANE	0.2
β-CARYOPHYLLENE 0.001	1.358	FARNESENE 0.001	1.152	CEDRENE 0.001	1.32} 98	LONGIPINENE 0.001	0.835	FARNESENE 0.001	1.126
β-CARYOPHYLLENE 0.01	1.402	FARNESENE 0.01	1.023	CEDRENE 0.01	1.489	LONGIPINENE 0.01	0.897	FARNESENE 0.01	1.128

β-CARYOPHYLLENE 0.1	1.729	FARNESENE 0.1	0.056	CEDRENE 0.1	1.169	LONGIPINENE 0.1	1.305	FARNESENE 0.1	1.205
β-CARYOPHYLLENE 1	1.607	FARNESENE 1	1.378	CEDRENE 1	1.147	LONGIPINENE 1	1.947	FARNESENE 1	1.391
HEXANE	0.05	HEXANE	0.2	HEXANE	0.2	HEXANE	0.05	HEXANE	0.2
AROMANDRENE 0.001	1.356	TML 0.001	1.265	AROMANDRENE 0.001	1.126	AROMANDRENE 0.001	1.568	LONGIPINENE 0.001	0.892
AROMANDRENE 0.01	1.289	TML 0.01	1.665	AROMANDRENE 0.01	1.019	AROMANDRENE 0.01	1.378	LONGIPINENE 0.01	0.965
AROMANDRENE 0.1	1.308	TML 0.1	1.876	AROMANDRENE 0.1	1.091	AROMANDRENE 0.1	1.405	LONGIPINENE 0.1	1.236
AROMANDRENE 1	1.398	TML 1	2.055	AROMANDRENE 1	1.156	AROMANDRENE 1	1.532	LONGIPINENE 1	1.523
HEXANE	0.02	HEXANE	0.02	HEXANE	0.2	HEXANE	0.025	HEXANE	0.1
CEDRENE 0.001	1.189	β-CARYOPHYLLENE 0.001	1.305	β-CARYOPHYLLENE 0.001	1.125	LONGIFOLENE 0.001	1.452	AROMANDRENE 0.001	1.523
CEDRENE 0.01	1.269	β-CARYOPHYLLENE 0.01	1.412	β-CARYOPHYLLENE 0.01	1.205	LONGIFOLENE 0.01	1.356	AROMANDRENE 0.01	1.4895
CEDRENE 0.1	1.354	β-CARYOPHYLLENE 0.1	1.756	β-CARYOPHYLLENE 0.1	1.564	LONGIFOLENE 0.1	1.405	AROMANDRENE 0.1	1.5023
CEDRENE 1	1.369	β-CARYOPHYLLENE 1	1.612	β-CARYOPHYLLENE 1	1.409	LONGIFOLENE 1	1.489	AROMANDRENE 1	1.598
HEXANE	0.1	HEXANE	0	HEXANE	0.11	HEXANE	0.235	HEXANE	0.158
FARNESENE 0.001	1.325	LONGIPINENE 0.001	0.982	LONGIFOLENE 0.001	1.245	TML 0.001	1.213	β-CARYOPHYLLENE 0.001	1.526

FARNESENE 0.01	1.289	LONGIPINENE 0.01	1.085	LONGIFOLENE 0.01	1.165	TML 0.01	1.325	β-CARYOPHYLLENE 0.01	1.623
FARNESENE 0.1	1.301	LONGIPINENE 0.1	1.329	LONGIFOLENE 0.1	0.998	TML 0.1	1.589	β-CARYOPHYLLENE 0.1	1.985
FARNESENE 1	1.502	LONGIPINENE 1	1.528	LONGIFOLENE 1	1.112	TML 1	1.915	β-CARYOPHYLLENE 1	1.812
HEXANE	0.1	HEXANE	0.1	HEXANE	0.158	HEXANE	0.125	HEXANE	0.165
TML 0.001	1.355	CEDRENE 0.001	0.985	LONGIPINENE 0.001	0.963	CEDRENE 0.001	1.312	LONGIFOLENE 0.001	1.402
TML 0.01	1.56	CEDRENE 0.01	1.06	LONGIPINENE 0.01	0.987	CEDRENE 0.01	1.451	LONGIFOLENE 0.01	1.265
TML 0.1	1.783	CEDRENE 0.1	1.561	LONGIPINENE 0.1	1.256	CEDRENE 0.1	1.569	LONGIFOLENE 0.1	1.307
TML 1	1.99	CEDRENE 1	1.578	LONGIPINENE 1	1.608	CEDRENE 1	1.502	LONGIFOLENE 1	1.409
REP 6		REP 7		REP 8		REP 9		REP 10	
HEXANE	0.023	HEXANE	0.012	HEXANE	0.278	HEXANE	0.156	HEXANE	0.064
FARNESENE 0.001	1.52	LONGIFOLENE 0.001	1.277	FARNESENE 0.001	1.305	β-CARYOPHYLLENE 0.001	1.506	CEDRENE 0.001	1.265
FARNESENE 0.01	1.423	LONGIFOLENE 0.01	1.456	FARNESENE 0.01	1.213	β-CARYOPHYLLENE 0.01	1.619	CEDRENE 0.01	1.369
FARNESENE 0.1	1.089	LONGIFOLENE 0.1	1.356	FARNESENE 0.1	1.308	β-CARYOPHYLLENE 0.1	1.91	CEDRENE 0.1	1.459
FARNESENE 1	1.318	LONGIFOLENE 1	1.423	FARNESENE 1	1.516	β-CARYOPHYLLENE 1	1.813	CEDRENE 1	1.499
HEXANE	0.125	HEXANE	0.036	HEXANE	0.1	HEXANE	0.147	HEXANE	0.087

AROMANDRENE 0.001	1.12	CEDRENE 0.001	0.982	TML 0.001	1.387	LONGIFOLENE 0.001	0.992	TML 0.001	1.002
AROMANDRENE 0.01	1.09	CEDRENE 0.01	1.034	TML 0.01	1.501	LONGIFOLENE 0.01	0.987	TML 0.01	1.489
AROMANDRENE 0.1	1.198	CEDRENE 0.1	1.182	TML 0.1	1.502	LONGIFOLENE 0.1	1.356	TML 0.1	1.702
AROMANDRENE 1	1.521	CEDRENE 1	1.193	TML 1	1.702	LONGIFOLENE 1	1.428	TML 1	1.751
HEXANE	0.1236	HEXANE	0.189	HEXANE	0.089	HEXANE	0.178	HEXANE	0.105
LONGIFOLENE 0.001	1.001	β-CARYOPHYLLENE 0.001	1.125	LONGIPINENE 0.001	0.798	AROMANDRENE 0.001	1.235	FARNESENE 0.001	1
LONGIFOLENE 0.01	0.958	β-CARYOPHYLLENE 0.01	1.298	LONGIPINENE 0.01	1.001	AROMANDRENE 0.01	1.125	FARNESENE 0.01	519
LONGIFOLENE 0.1	1.005	β-CARYOPHYLLENE 0.1	1.513	LONGIPINENE 0.1	1.198	AROMANDRENE 0.1	1.302	FARNESENE 0.1	1.406
LONGIFOLENE 1	1.091	β-CARYOPHYLLENE 1	1.436	LONGIPINENE 1	1.492	AROMANDRENE 1	1.555	FARNESENE 1	1.458
HEXANO	0.202	HEXANE	0.165	HEXANE	0.012	HEXANE	0.158	HEXANE	1.705
TML 0.001	1.358	AROMANDRENE 0.001	1.102	AROMANDRENE 0.001	1.352	CEDRENE 0.001	1.325	LONGIPINENE 0.001	0.752
TML 0.01	1.698	AROMANDRENE 0.01	1.025	AROMANDRENE 0.01	1.253	CEDRENE 0.01	1.418	LONGIPINENE 0.01	0.856
TML 0.1	1.792	AROMANDRENE 0.1	1.09	AROMANDRENE 0.1	1.301	CEDRENE 0.1	1.183	LONGIPINENE 0.1	1.64
TML 1	2.009	AROMANDRENE 1	1.28	AROMANDRENE 1	1.509	CEDRENE 1	1.199	LONGIPINENE 1	1.85
HEXANE	0.258	HEXANE	0.256	HEXANE	0.253	HEXANE	0.65	HEXANE	0.02

β-CARYOPHYLLENE 0.001	1.564	LONGIPINENE 0.001	1.018	β-CARYOPHYLLENE 0.001	1.205	TML 0.001	1.258	AROMADENDRENE 0.001	1.305
β-CARYOPHYLLENE 0.01	1.698	LONGIPINENE 0.01	1.169	β-CARYOPHYLLENE 0.01	1.298	TML 0.01	1.603	AROMADENDRENE 0.01	1.236
β-CARYOPHYLLENE 0.1	1.915	LONGIPINENE 0.1	1.3	β-CARYOPHYLLENE 0.1	1.562	TML 0.1	1.728	AROMADENDRENE 0.1	1.269
β-CARYOPHYLLENE 1	1.628	LONGIPINENE 1	1.258	β-CARYOPHYLLENE 1	1.447	TML 1	2.094	AROMADENDRENE 1	1.361
HEXANE	0.214	HEXANE	0.3	HEXANE	0.025	HEXANE	0.014	HEXANE	0.58
CEDRENE 0.001	1.152	TML 0.001	1.269	CEDRENE 0.001	1.158	LONGIPINENE 0.001	1.098	β-CARYOPHYLLENE 0.001	1.369
CEDRENE 0.01	1.236	TML 0.01	1.46	CEDRENE 0.01	1.236	LONGIPINENE 0.01	1.098	β-CARYOPHYLLENE 0.01	1.473
CEDRENE 0.1	1.387	TML 0.1	1.628	CEDRENE 0.1	1.31	LONGIPINENE 0.1	1.201	β-CARYOPHYLLENE 0.1	1.702
CEDRENE 1	1.395	TML 1	1.914	CEDRENE 1	1.393	LONGIPINENE 1	1.564	β-CARYOPHYLLENE 1	1.596
HEXANE	0.124	HEXANE	0.289	HEXANE	0.236	HEXANE	0.023	HEXANE	0.098
LONGIPINENE 0.001	0.745	FARNESENE 0.001	1.256	LONGIFOLENE 0.001	1.098	FARNESENE 0.001	1.136	LONGIFOLENE 0.001	1.309
LONGIPINENE 0.01	1.056	FARNESENE 0.01	1.125	LONGIFOLENE 0.01	1.089	FARNESENE 0.01	1.078	LONGIFOLENE 0.01	1.256
LONGIPINENE 0.1	1.263	FARNESENE 0.1	1.98	LONGIFOLENE 0.1	1.125	FARNESENE 0.1	1.109	LONGIFOLENE 0.1	0.985
LONGIPINENE 1	1.603	FARNESENE 1	1.394	LONGIFOLENE 1	1.456	FARNESENE 1	1.319	LONGIFOLENE 1	1.123
